# Supplementary material for: Modelling armed conflict risk under climate change with machine learning and time-series data
Source: Nat Commun. 2022 May 20;13:2839. doi: 10.1038/s41467-022-30356-x (PMC9123163; doi:10.1038/s41467-022-30356-x)
Supplement: Supplementary file 1 — Supplementary Information [file 41467_2022_30356_MOESM1_ESM.pdf]

Supplementary Information

For

Modelling armed conflict risk under climate change with machine learning and high-frequency time-series data

Quansheng Ge[1,8], Mengmeng Hao[1,2,8], Fangyu Ding[1,2,*], Dong Jiang[1,2,3,*], Jürgen Scheffran[4], David Helman[5,6], Tobias Ide[7]

[1]Institute of Geographic Sciences and Natural Resources Research, Chinese Academy of Sciences, Beijing 100101, China.

[2]College of Resources and Environment, University of Chinese Academy of Sciences, Beijing 100049, China.

[3]Key Laboratory of Carrying Capacity Assessment for Resource and Environment, Ministry of Land & Resources, Beijing 100101, China.

[4]Institute of Geography, Center for Earth System Research and Sustainability, University of Hamburg, Hamburg 20144, Germany.

[5]Institute of Environmental Sciences, Department of Soil and Water Sciences, The Robert H. Smith Faculty of Agriculture, Food & Environment, The Hebrew University of Jerusalem, Rehovot 7610001, Israel.

[6]Advanced School for Environmental Studies, The Hebrew University of Jerusalem, Jerusalem, Israel.

[7]Centre for Biosecurity and One Health, Harry Butler Institute, Murdoch University, Murdoch 6150, Perth, Australia.

[8]These authors contribute equally: Quansheng Ge, Mengmeng Hao.

[*]Correspondence and requests for materials should be addressed to: Fangyu Ding (dingfy.17b@igsnrr.ac.cn) and Dong Jiang (jiangd@igsnrr.ac.cn)

Content

Supplementary Figures 3
Supplementary Tables 20
Supplementary Notes 23
Dependent Variable 23
Independent Variable 24
Supplementary Methods 25
Boosted Regression Trees 25
BRT Parameter Selection 26
Modelling Strategy 26
Statistical Test 27
Supplementary References 34

Supplementary Figures

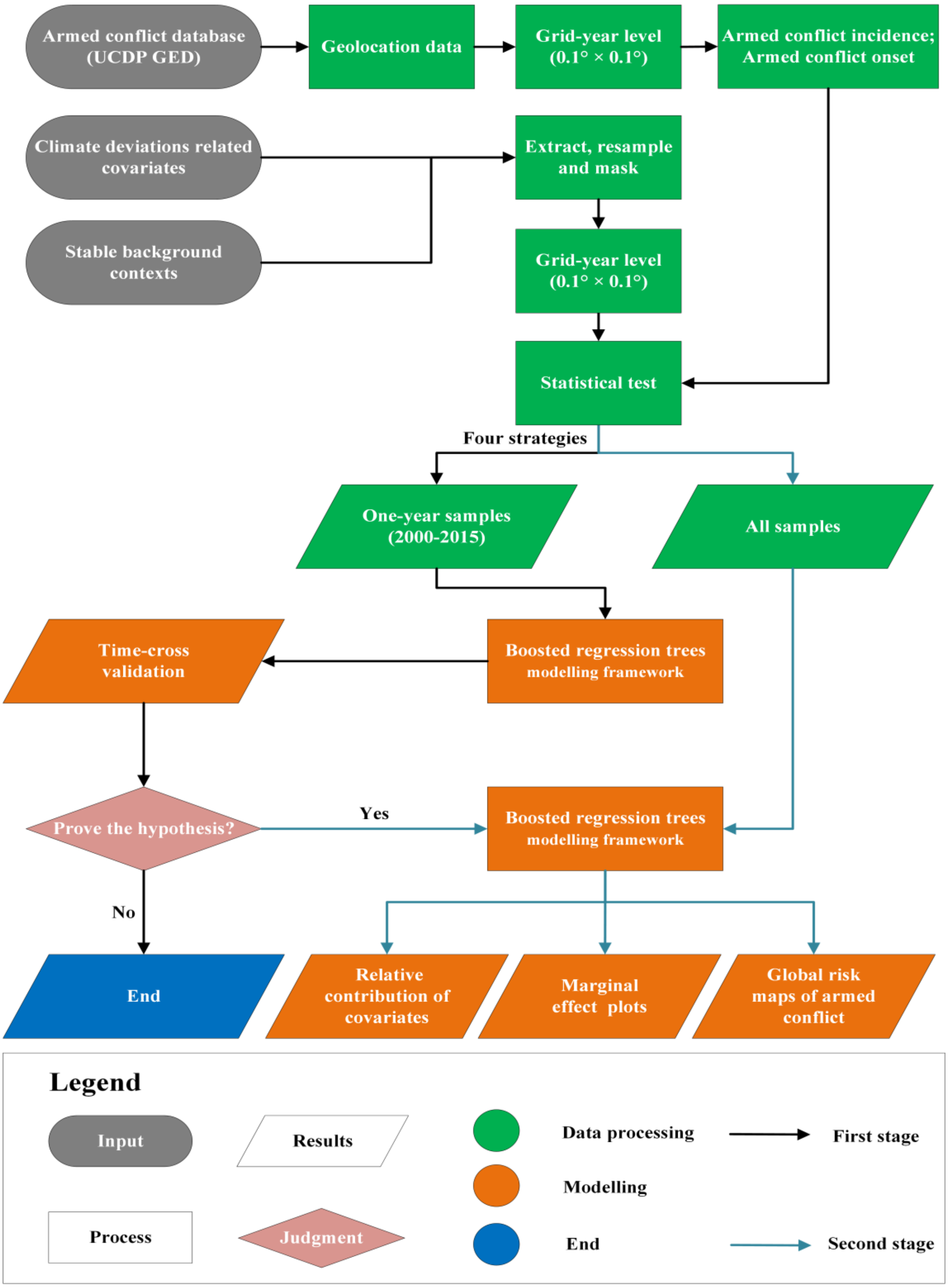

Supplementary Figure 1. Analytic process overview. The process used to simulate the risk of armed conflict at global scale involved two stages. In the first stage (black arrow), the input dataset was combined with the BRT modelling framework to prove the hypothesis. If the hypothesis was proved to be true, the second stage (blue arrow) would start the analysis. Otherwise the analytic process would end.

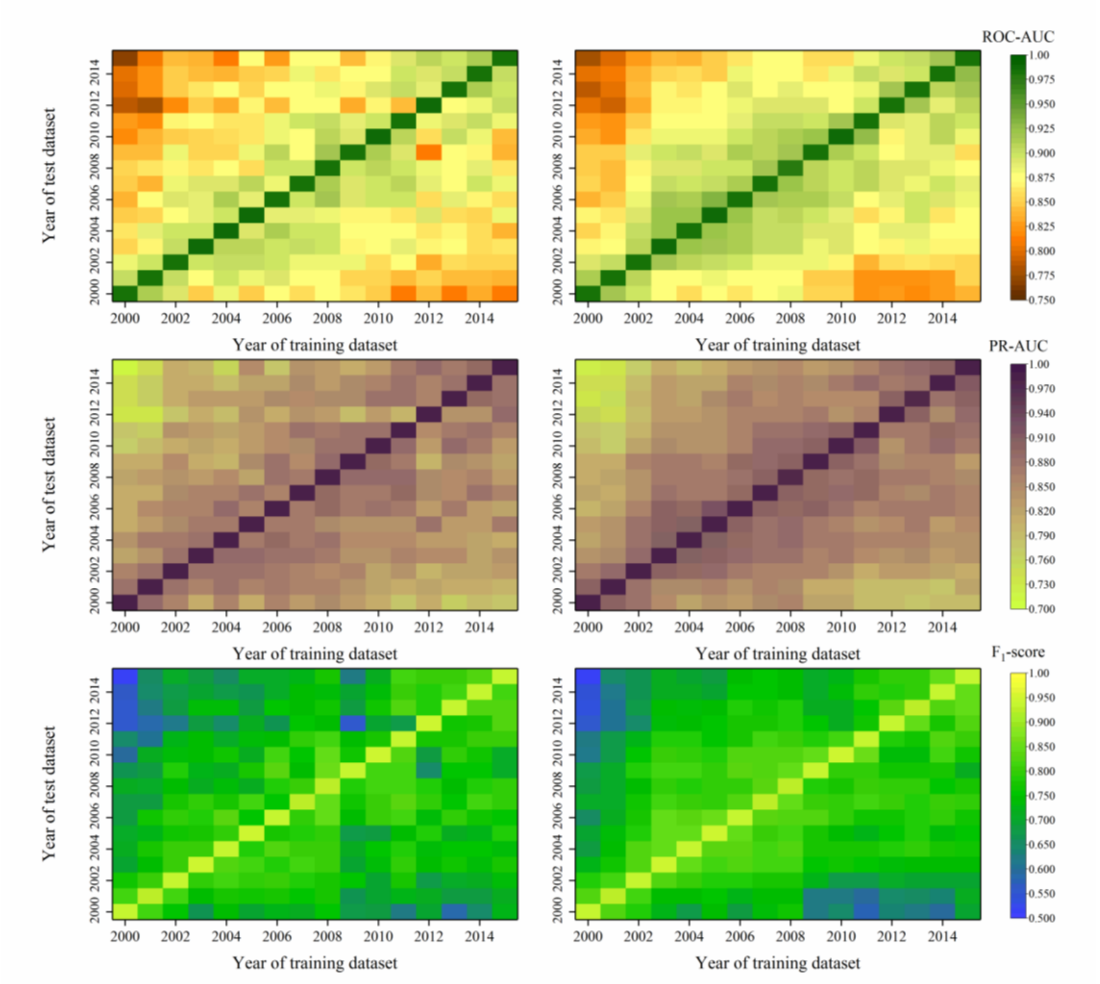

Supplementary Figure 2. Validation performance on a time scale of the BRT models trained on one-year incidence samples. Validation performance of strategies a and a+ are shown in the left and right columns, respectively.

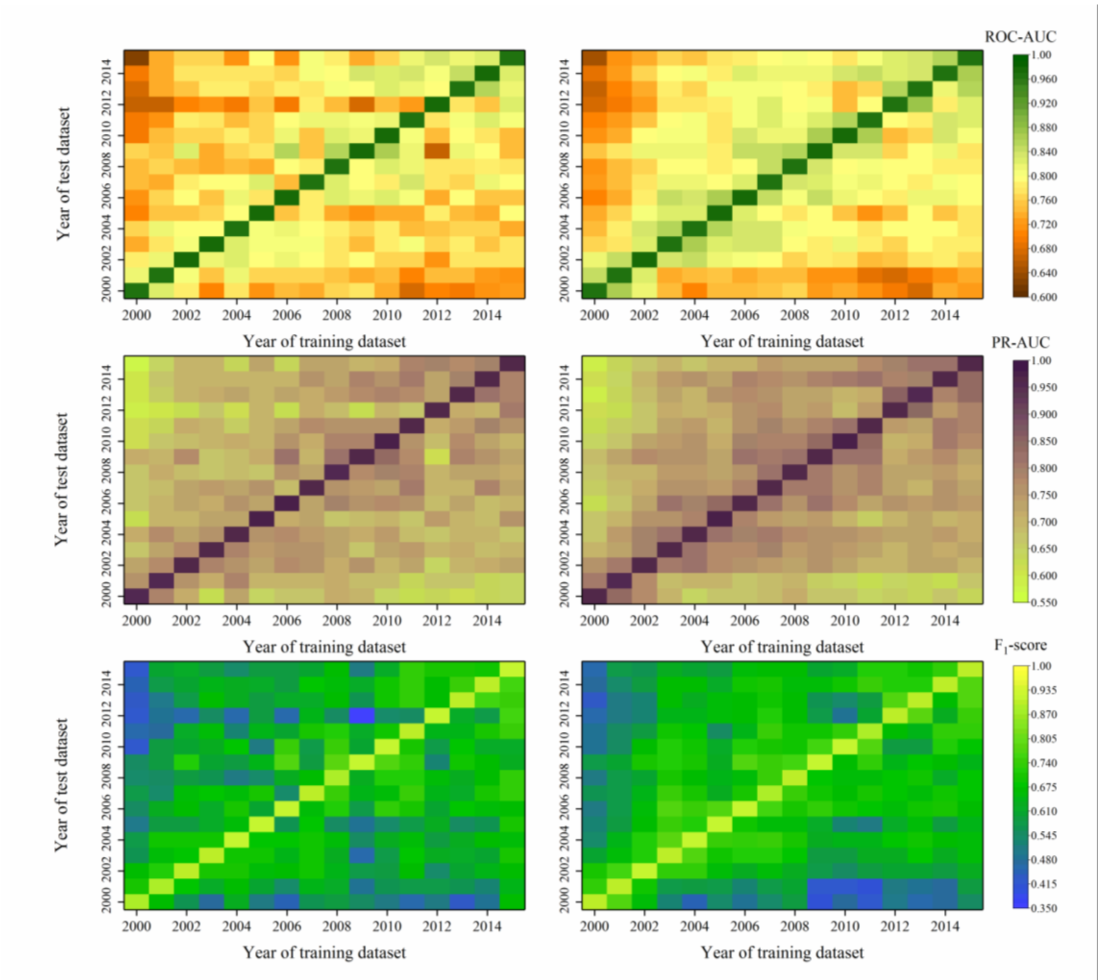

Supplementary Figure 3. Validation performance on a time scale of the BRT models trained on one-year incidence samples. Validation performance of strategies b and b+ are shown in the left and right columns, respectively.

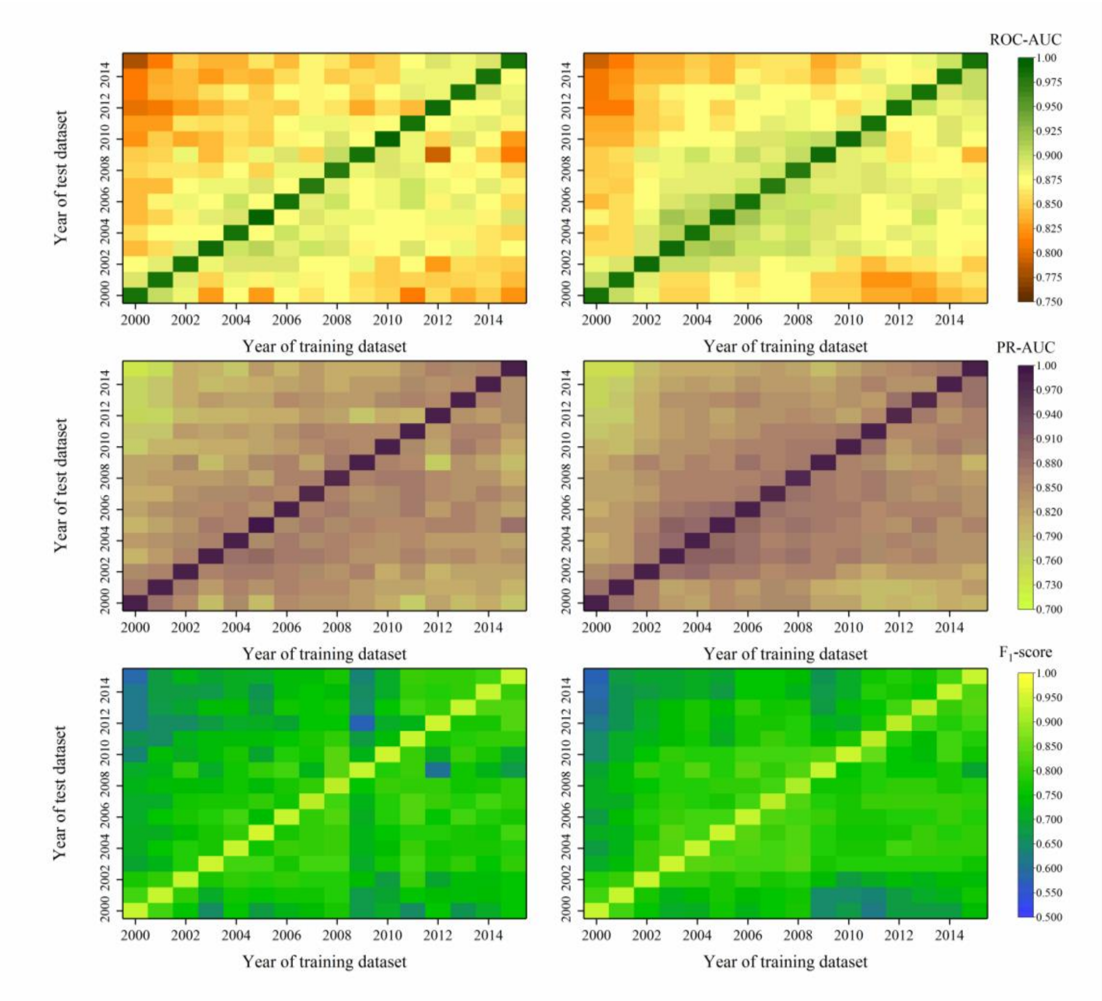

Supplementary Figure 4. Validation performance on a time scale of the BRT models trained on one-year onset samples. Validation performance of strategies a and a+ are shown in the left and right columns, respectively.

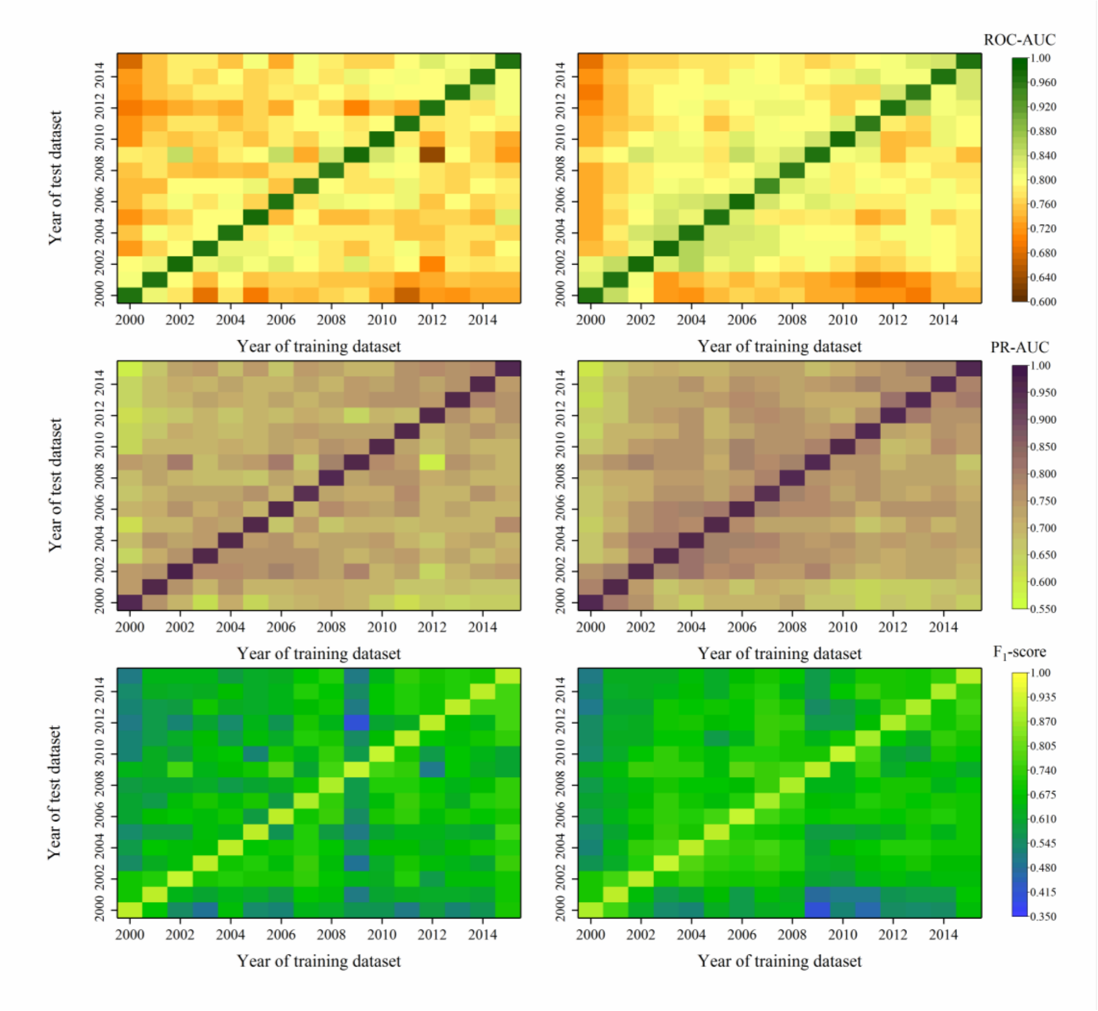

Supplementary Figure 5. Validation performance on a time scale of the BRT models trained on one-year onset samples. Validation performance of strategies b and b+ are shown in the left and right columns, respectively.

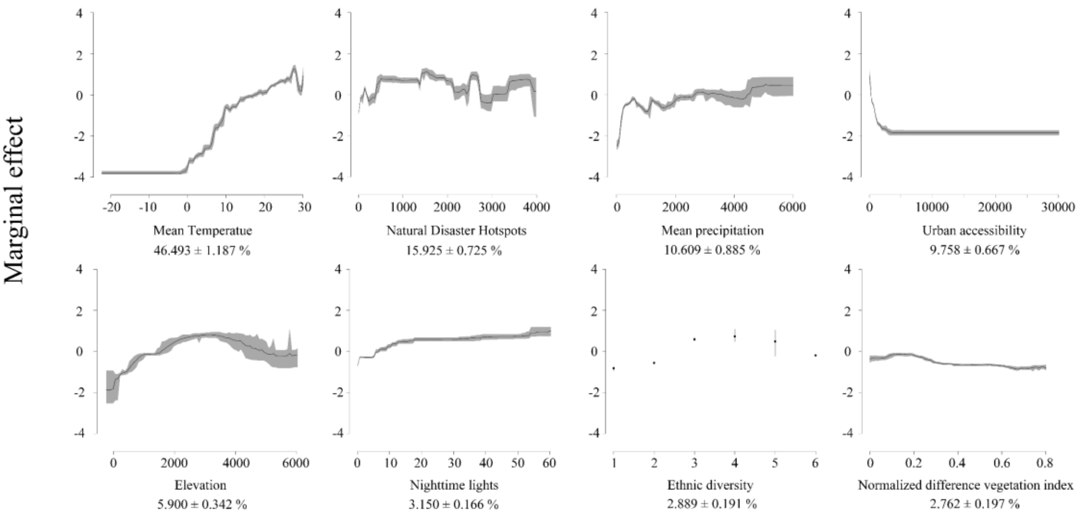

Supplementary Figure 6. Marginal effect curves of each stable background covariate over the BRT ensembles fitted to the full incidence samples under strategy a+. The black lines represent the mean effect curves calculated from the ensemble BRT models and the dark grey the 95% confidence interval. Sub-plots are ordered by the mean relative contribution (%) of covariates, with these mean relative contribution ± standard deviation (%) given within each sub-plot.

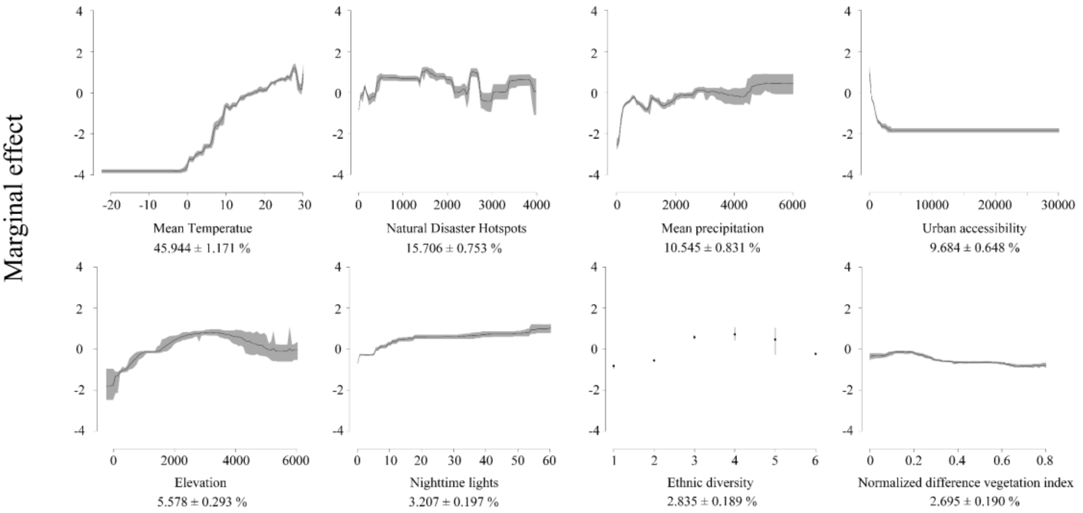

Supplementary Figure 7. Marginal effect curves of each stable background covariate over the BRT ensembles fitted to the full incidence samples under strategy a. The black lines represent the mean effect curves calculated from the ensemble BRT models and the dark grey the 95% confidence interval. Sub-plots are ordered by the mean relative contribution (%) of covariates, with these mean relative contribution ± standard deviation (%) given within each sub-plot.

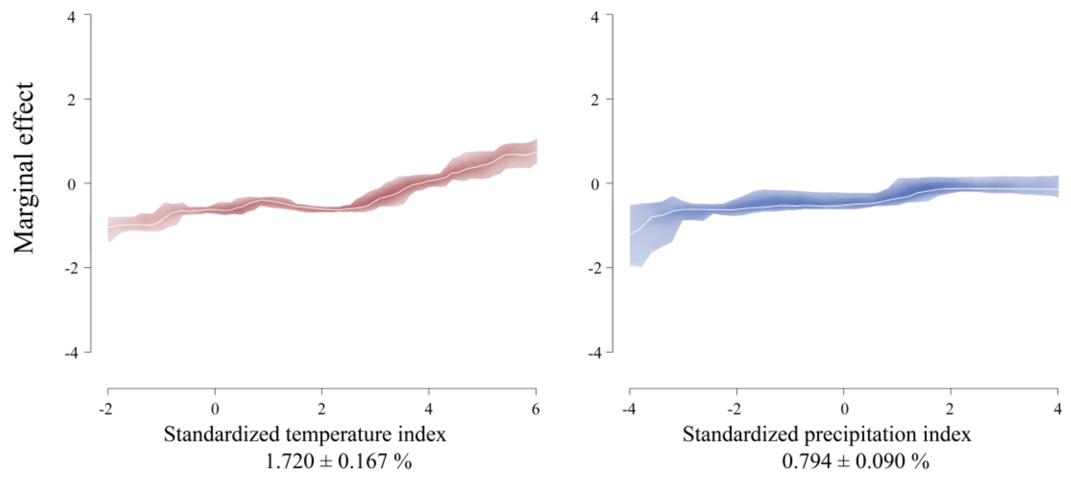

Supplementary Figure 8. Marginal effect curves of each climate deviations related covariate over the BRT ensembles fitted to the full incidence samples under strategy a. The white lines represent the mean effect curves calculated from the ensemble BRT models. 95% confidence interval of climate variables are indicated by color: red, standardized temperature index; blue, Standardized precipitation index. Sub-plots are ordered by the mean relative contribution (%) of covariates, with these mean relative contribution ± standard deviation (%) given within each sub-plot.

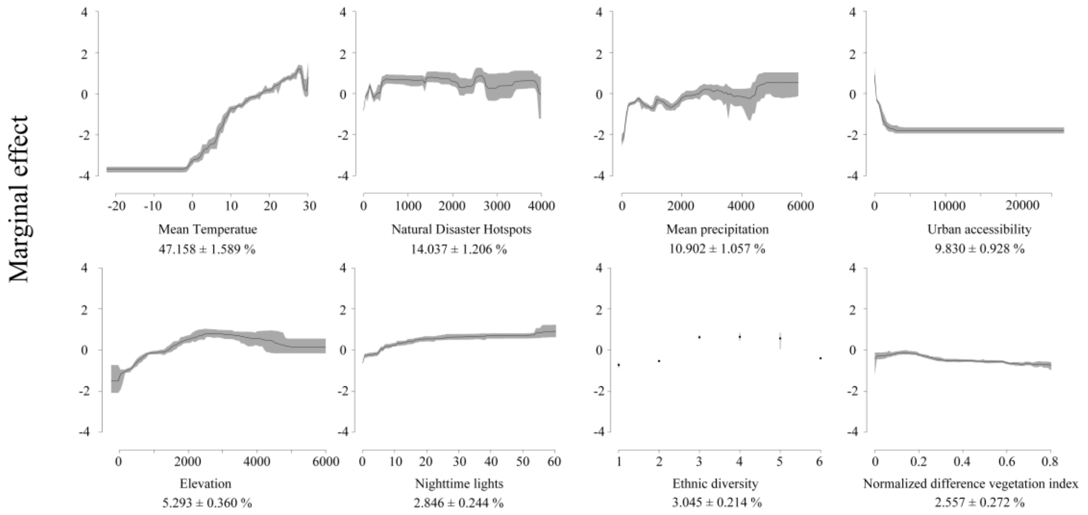

Supplementary Figure 9. Marginal effect curves of each stable background covariate over the BRT ensembles fitted to the full onset samples under strategy a+. The black lines represent the mean effect curves calculated from the ensemble BRT models and the dark grey the 95% confidence interval. Sub-plots are ordered by the mean relative contribution (%) of covariates, with these mean relative contribution ± standard deviation (%) given within each sub-plot.

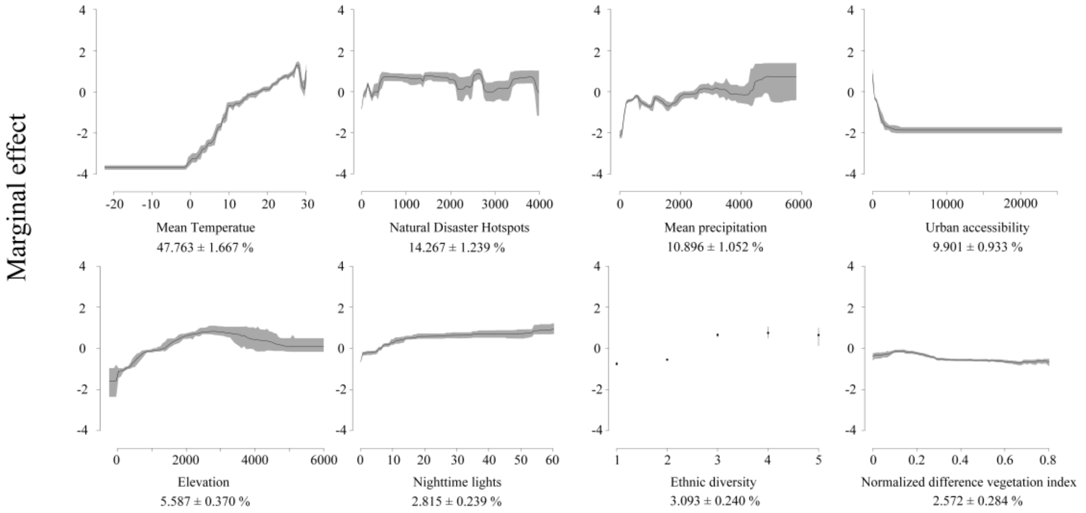

Supplementary Figure 10. Marginal effect curves of each stable background covariate over the BRT ensembles fitted to the full onset samples under strategy a. The black lines represent the mean effect curves calculated from the ensemble BRT models and the dark grey the 95% confidence interval. Sub-plots are ordered by the mean relative contribution (%) of covariates, with these mean relative contribution ± standard deviation (%) given within each sub-plot.

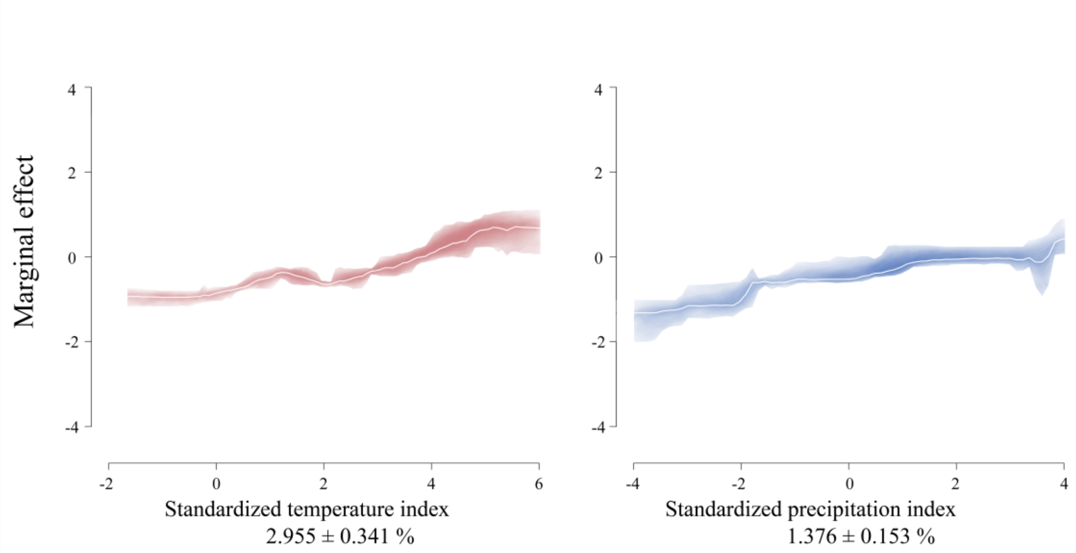

Supplementary Figure 11. Marginal effect curves of each climate deviation related covariate over the BRT ensembles fitted to the full onset samples under strategy a+. The white lines represent the mean effect curves calculated from the ensemble BRT models. 95% confidence interval of climate variables are indicated by color: red, standardized temperature index; blue, Standardized precipitation index. Sub-plots are ordered by the mean relative contribution (%) of covariates, with these mean relative contribution ± standard deviation (%) given within each sub-plot.

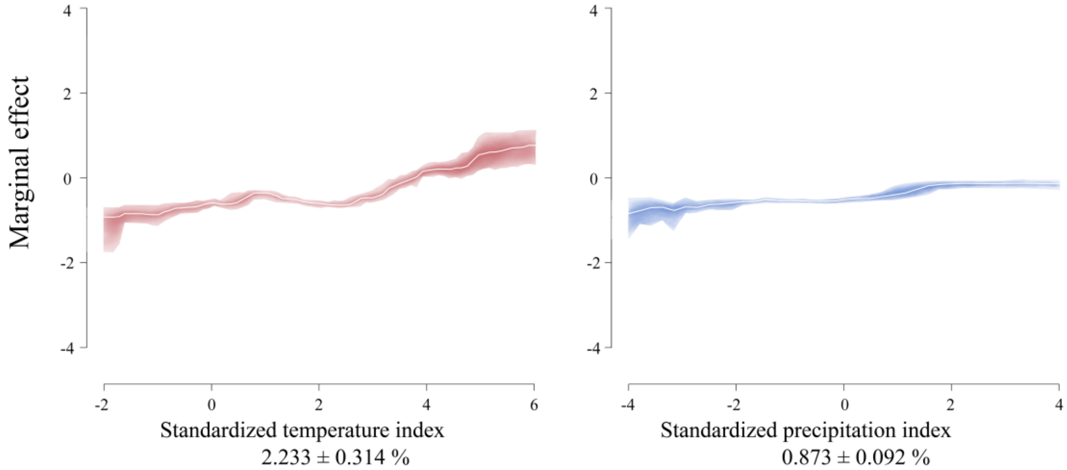

Supplementary Figure 12. Marginal effect curves of each climate deviation related covariate over the BRT ensembles fitted to the full onset samples under strategy a. The white lines represent the mean effect curves calculated from the ensemble BRT models. 95% confidence interval of climate variables are indicated by color: red, standardized temperature index; blue, Standardized precipitation index. Sub-plots are ordered by the mean relative contribution (%) of covariates, with these mean relative contribution ± standard deviation (%) given within each sub-plot.

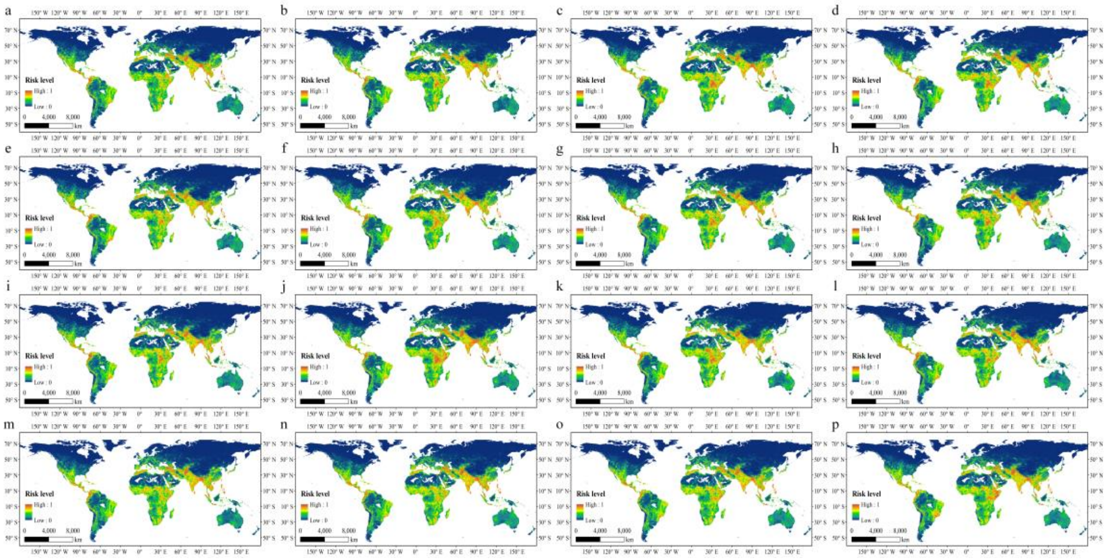

Supplementary Figure 13. Maps of the global simulated risk of armed conflict incidence at 0.1° × 0.1° spatial resolution based on 20 ensemble BRT models trained on all incidence samples under strategy a. The simulated risk level ranges from 0 (blue) to 1 (red).

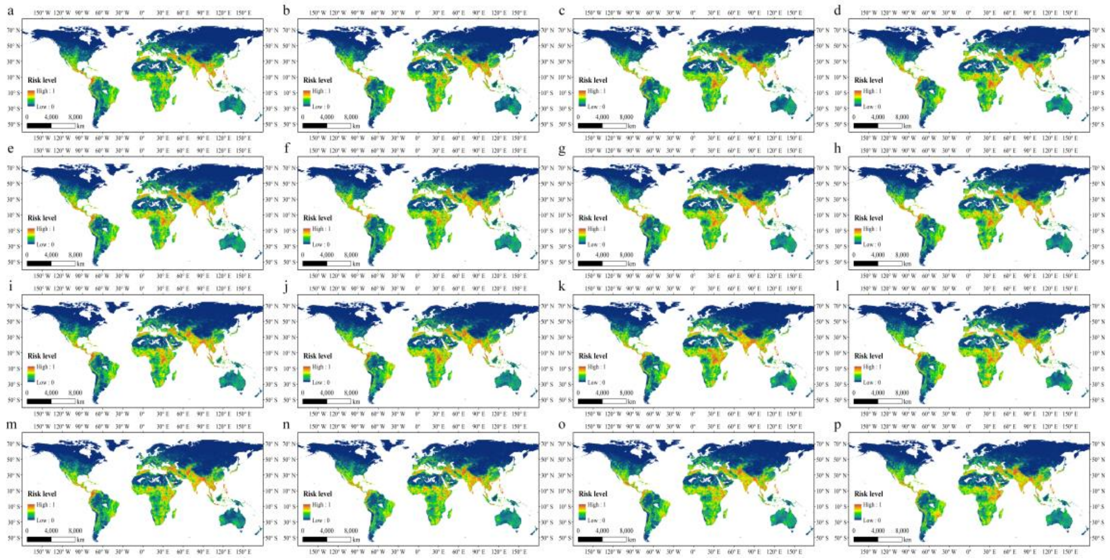

Supplementary Figure 14. Maps of the global simulated risk of armed conflict incidence at 0.1° × 0.1° spatial resolution based on 20 ensemble BRT models trained on all incidence samples under strategy a+. The simulated risk level ranges from 0 (blue) to 1 (red).

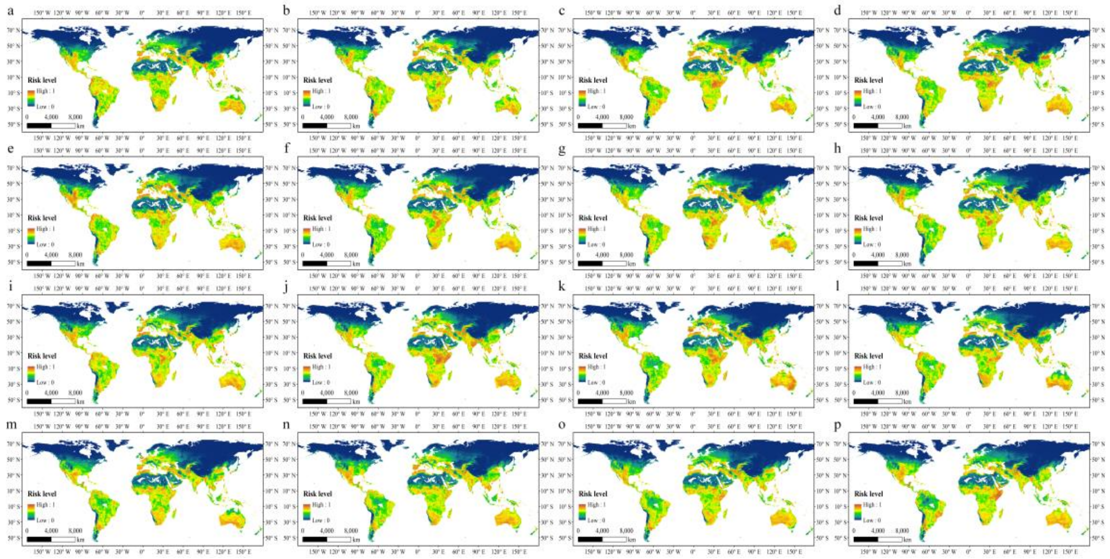

Supplementary Figure 15. Maps of the global simulated risk of armed conflict incidence at 0.1° × 0.1° spatial resolution based on 20 ensemble BRT models trained on all incidence samples under strategy b. The simulated risk level ranges from 0 (blue) to 1 (red).

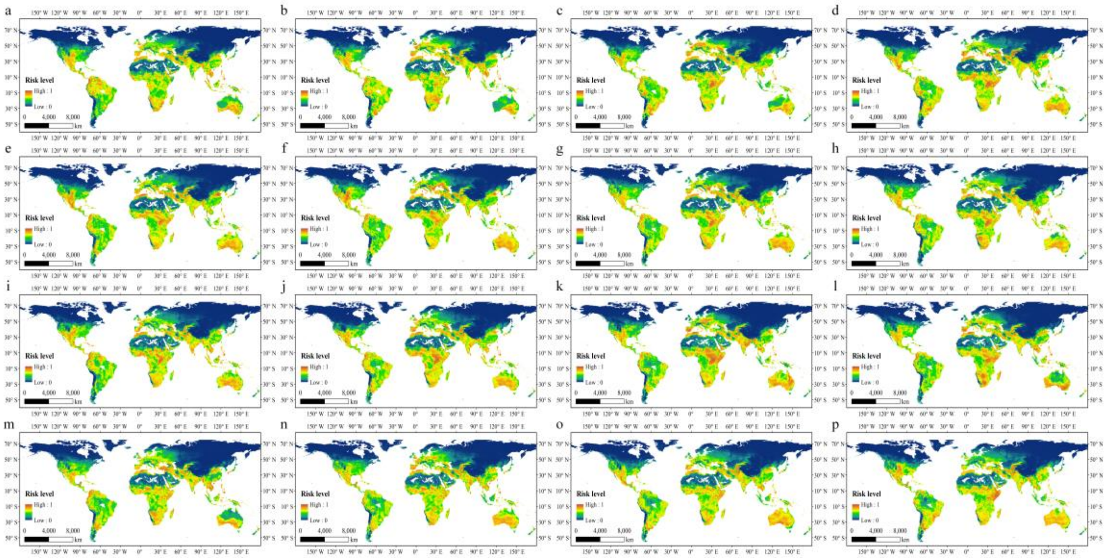

Supplementary Figure 16. Maps of the global simulated risk of armed conflict incidence at 0.1° × 0.1° spatial resolution based on 20 ensemble BRT models trained on all incidence samples under strategy b+. The simulated risk level ranges from 0 (blue) to 1 (red).

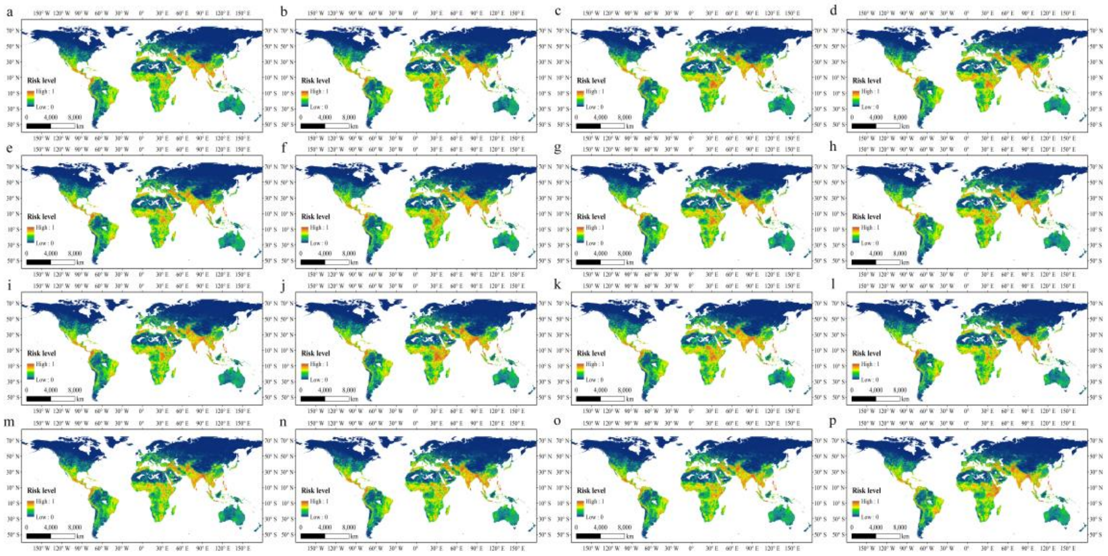

Supplementary Figure 17. Maps of the global simulated risk of armed conflict onset at 0.1° × 0.1° spatial resolution based on 20 ensemble BRT models trained on all onset samples under strategy a. The simulated risk level ranges from 0 (blue) to 1 (red).

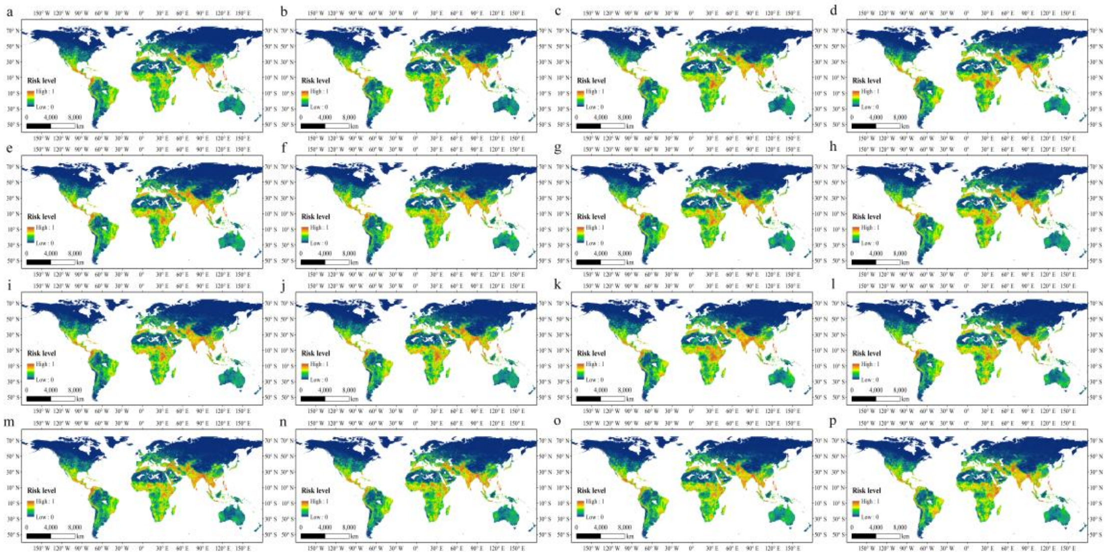

Supplementary Figure 18. Maps of the global simulated risk of armed conflict onset at 0.1° × 0.1° spatial resolution based on 20 ensemble BRT models trained on all onset samples under strategy a+. The simulated risk level ranges from 0 (blue) to 1 (red).

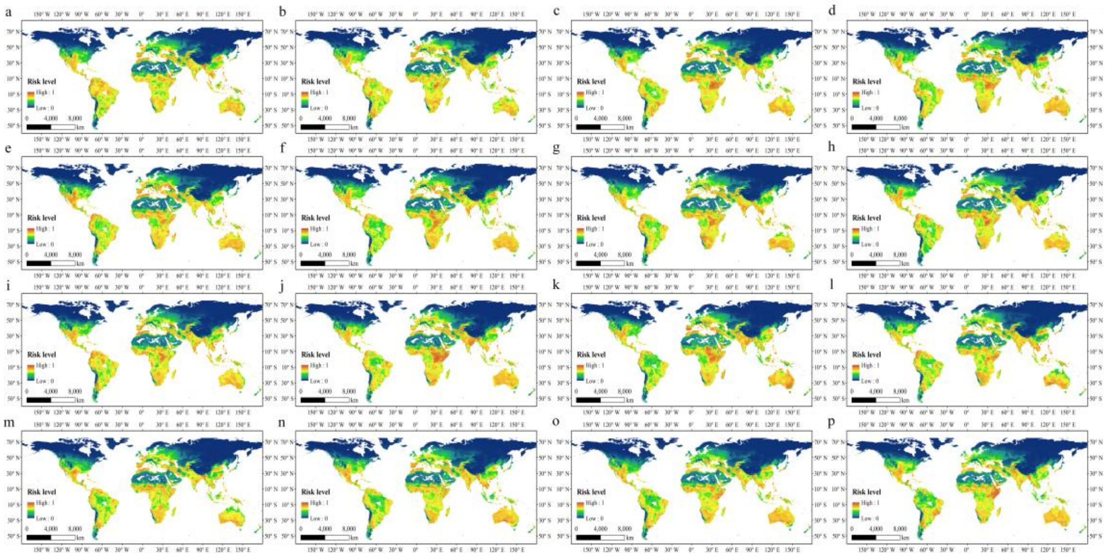

Supplementary Figure 19. Maps of the global simulated risk of armed conflict onset at 0.1° × 0.1° spatial resolution based on 20 ensemble BRT models trained on all onset samples under strategy b. The simulated risk level ranges from 0 (blue) to 1 (red).

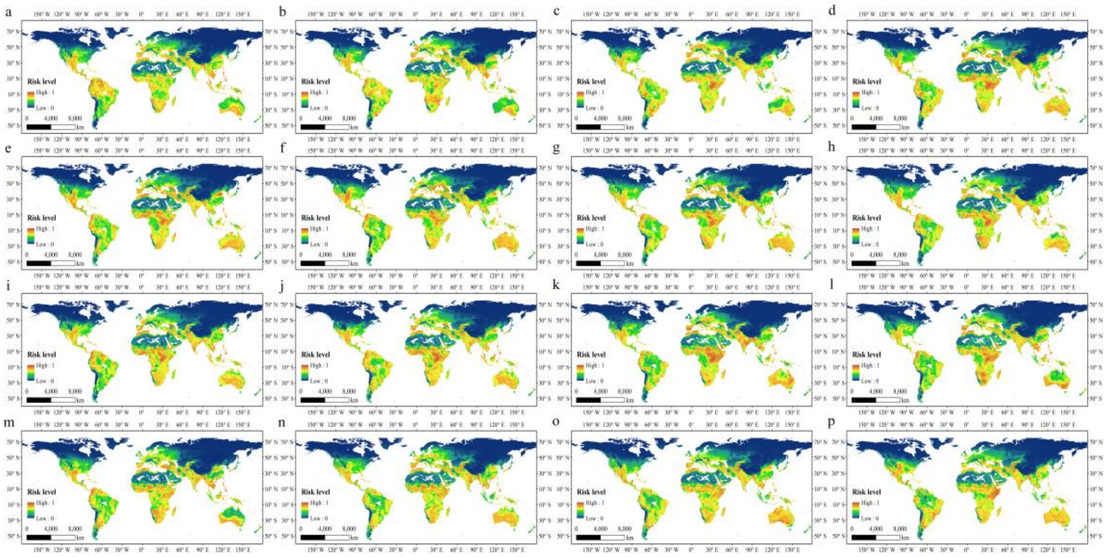

Supplementary Figure 20. Maps of the global simulated risk of armed conflict onset at 0.1° × 0.1° spatial resolution based on 20 ensemble BRT models trained on all onset samples under strategy b+. The simulated risk level ranges from 0 (blue) to 1 (red).

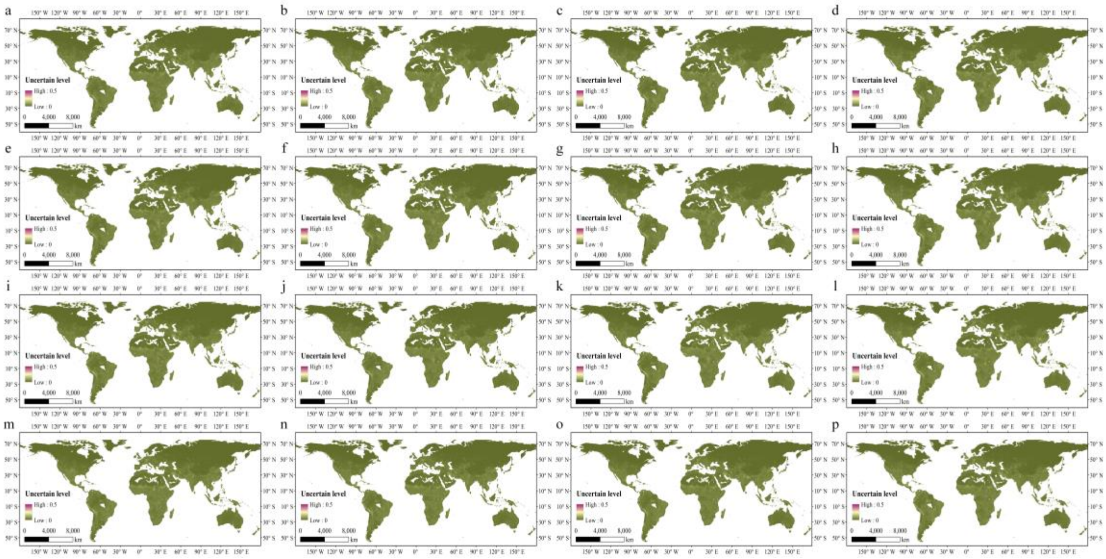

Supplementary Figure 21. Maps of uncertainty associated with these simulations derived from 20 ensemble BRT models trained on all incidence samples under strategy a.

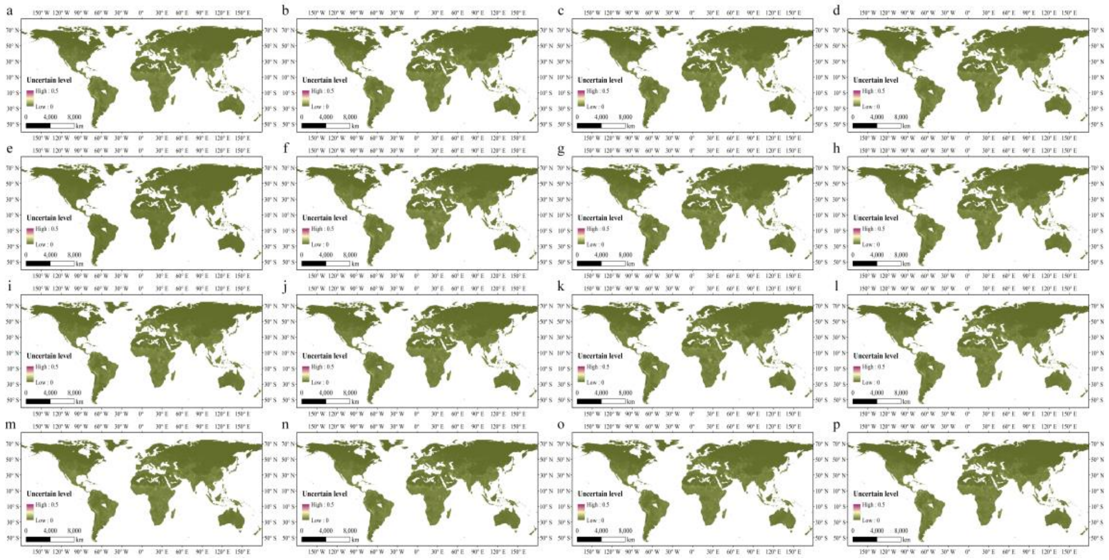

Supplementary Figure 22. Maps of uncertainty associated with these simulations derived from 20 ensemble BRT models trained on all incidence samples under strategy a+.

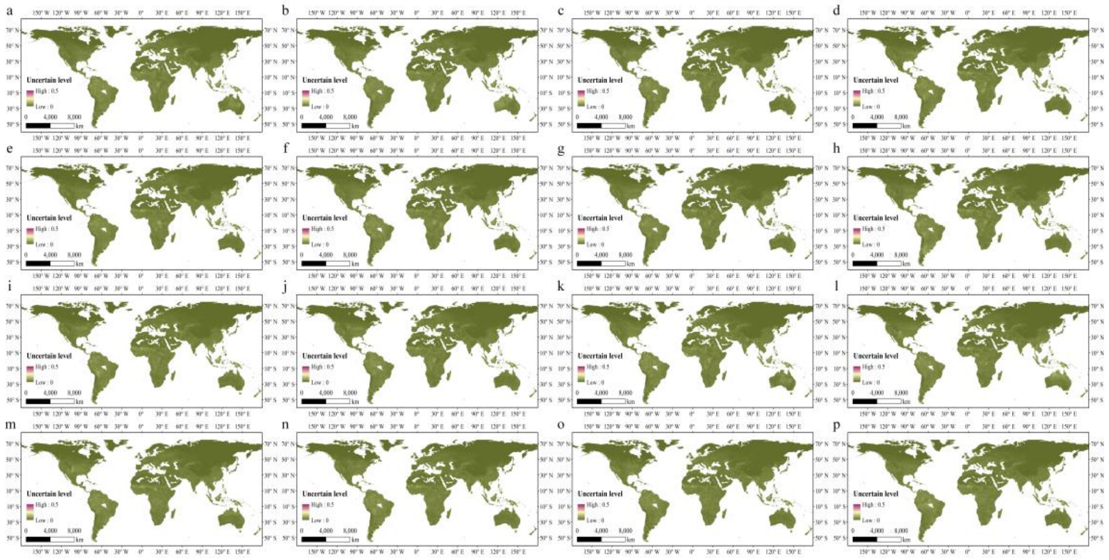

Supplementary Figure 23. Maps of uncertainty associated with these simulations derived from 20 ensemble BRT models trained on all incidence samples under strategy b.

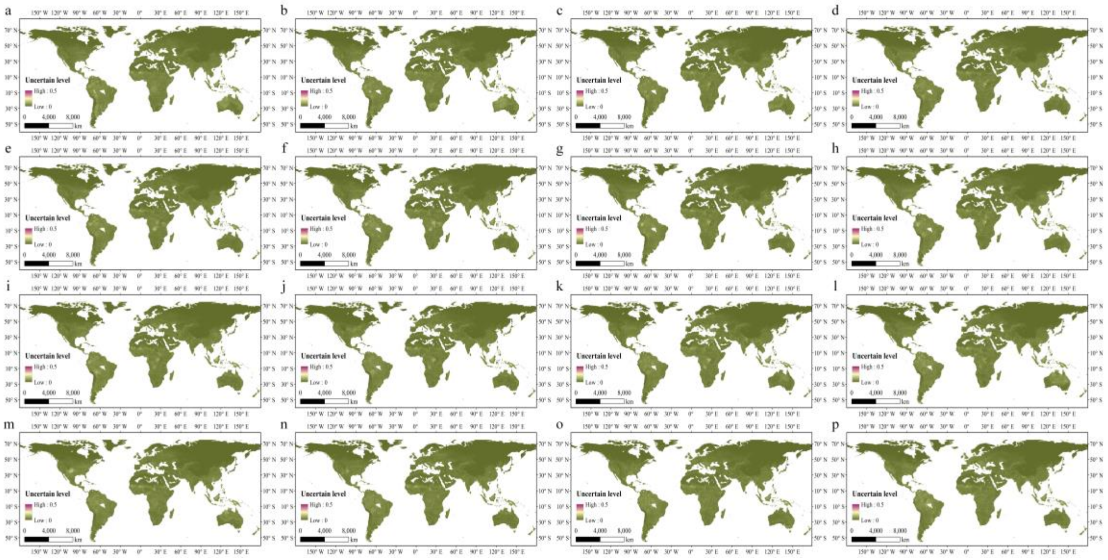

Supplementary Figure 24. Maps of uncertainty associated with these simulations derived from 20 ensemble BRT models trained on all incidence samples under strategy b+.

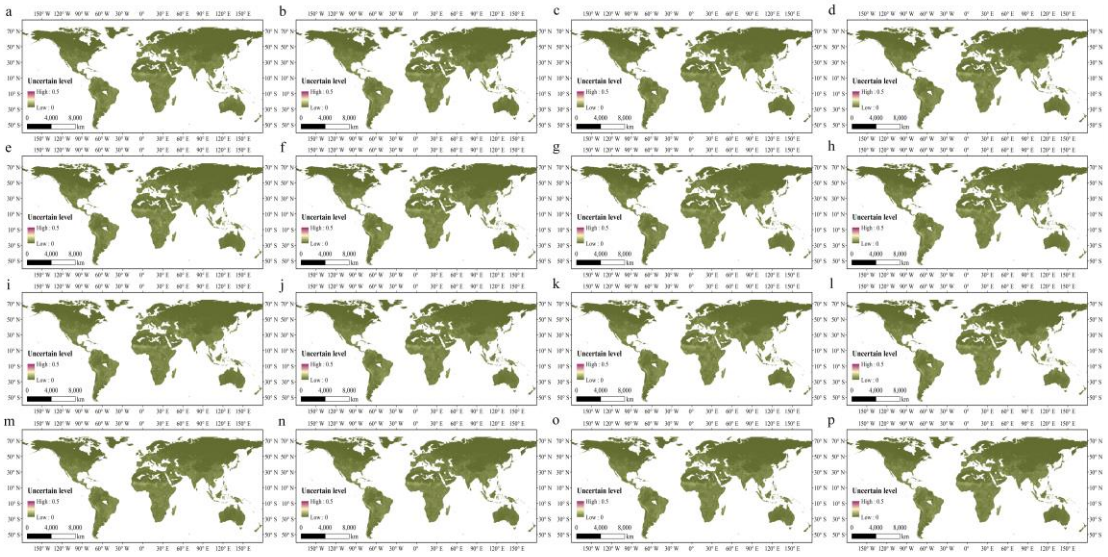

Supplementary Figure 25. Maps of uncertainty associated with these simulations derived from 20 ensemble BRT models trained on all onset samples under strategy a.

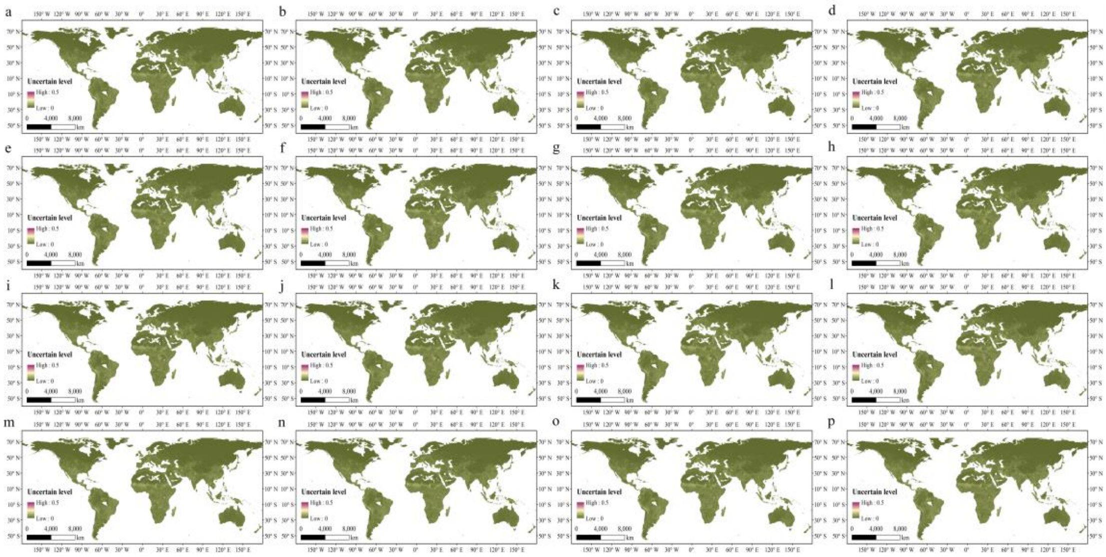

Supplementary Figure 26. Maps of uncertainty associated with these simulations derived from 20 ensemble BRT models trained on all onset samples under strategy a+.

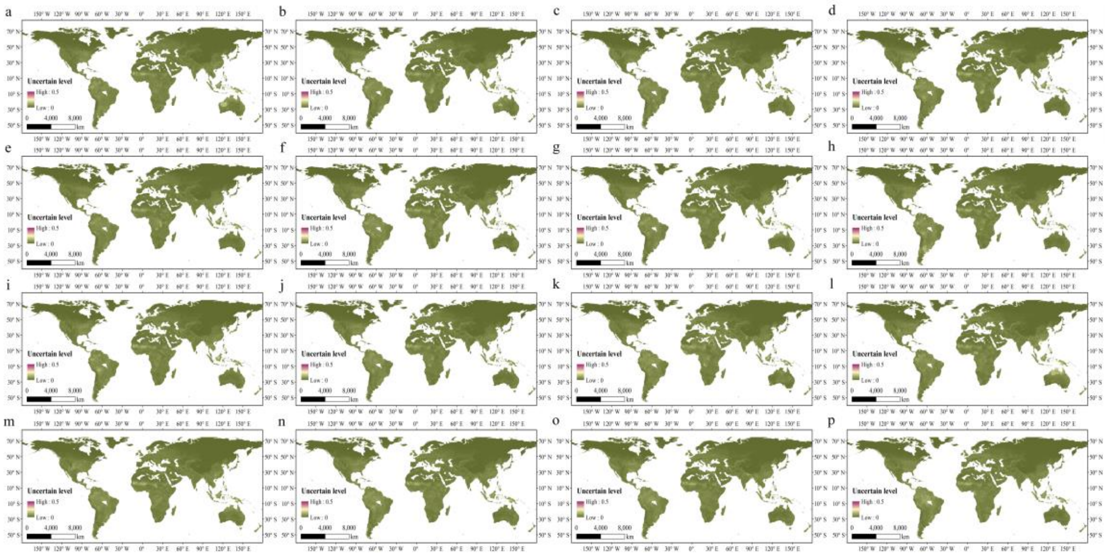

Supplementary Figure 27. Maps of uncertainty associated with these simulations derived from 20 ensemble BRT models trained on all onset samples under strategy b.

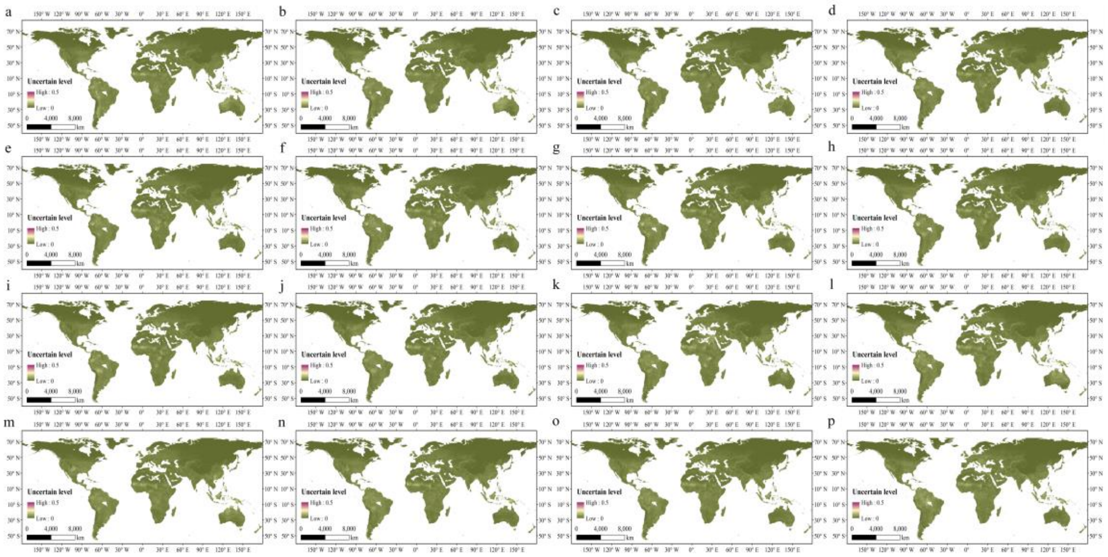

Supplementary Figure 28. Maps of uncertainty associated with these simulations derived from 20 ensemble BRT models trained on all onset samples under strategy b+.

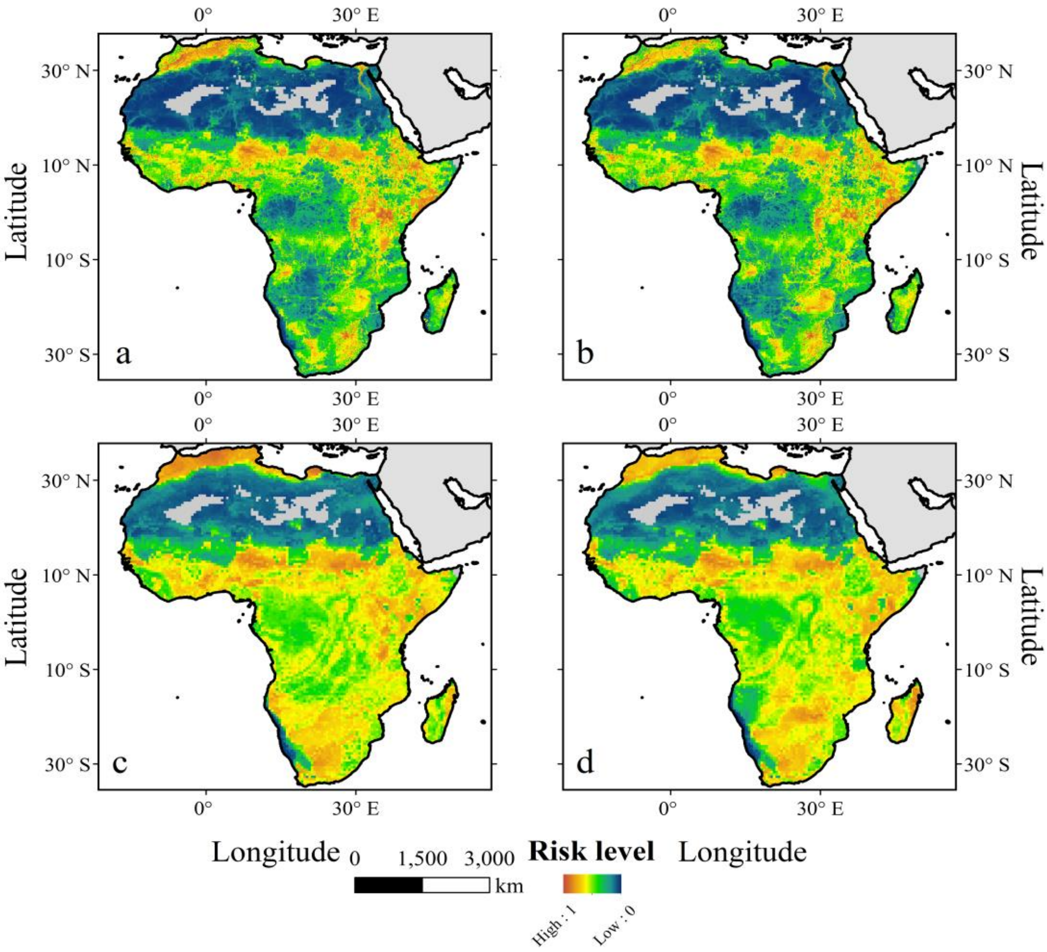

Supplementary Figure 29. Maps of the simulated risk of armed conflict incidence in Africa in 2018 are generated from 20 ensemble BRT models fitted from full incidence samples under strategies (a) a, (b) a+, (c) b, and (d) b+. The simulated risk level ranges from 0 (blue) to 1 (red), and the grey part denotes the areas with insufficient data.

Supplementary Tables

Supplementary Table 1. The performance of the 20 ensemble BRT models trained on one-year incidence samples during time-cross validation process.

Performance	Strategy a		Strategy a+		Strategy b		Strategy b+	
	Mean	Standard Deviation	Mean	Standard Deviation	Mean	Standard Deviation	Mean	Standard Deviation
ROC-AUC	0.878	0.038	0.886	0.039	0.784	0.062	0.798	0.061
PR-AUC	0.851	0.048	0.860	0.049	0.731	0.078	0.751	0.077
F_1-score	0.756	0.076	0.767	0.078	0.638	0.105	0.657	0.105

Supplementary Table 2. The performance of the 20 ensemble BRT models trained on one-year onset samples during time-cross validation process.

Performance	Strategy a		Strategy a+		Strategy b		Strategy b+	
	Mean	Standard Deviation	Mean	Standard Deviation	Mean	Standard Deviation	Mean	Standard Deviation
ROC-AUC	0.873	0.036	0.880	0.035	0.785	0.056	0.798	0.054
PR-AUC	0.842	0.045	0.851	0.044	0.731	0.071	0.749	0.068
F_1-score	0.762	0.067	0.771	0.066	0.661	0.090	0.677	0.086

Supplementary Table 3. The performance of the 20 ensemble BRT models trained on all incidence samples under different strategies.

Performance	Strategy a		Strategy a+		Strategy b		Strategy b+	
	Mean	Standard Deviation	Mean	Standard Deviation	Mean	Standard Deviation	Mean	Standard Deviation
ROC-AUC	0.937	0.001	0.939	0.002	0.886	0.003	0.891	0.002
PR-AUC	0.935	0.002	0.937	0.002	0.880	0.003	0.887	0.002
F_1-score	0.879	0.002	0.882	0.002	0.827	0.003	0.830	0.003

Supplementary Table 4. The performance of the 20 ensemble BRT models trained on all onset samples under different strategies.

Performance	Strategy a		Strategy a+		Strategy b		Strategy b+	
	Mean	Standard Deviation	Mean	Standard Deviation	Mean	Standard Deviation	Mean	Standard Deviation
ROC-AUC	0.927	0.002	0.928	0.002	0.868	0.004	0.874	0.004
PR-AUC	0.926	0.002	0.928	0.002	0.862	0.004	0.869	0.004
F_1-score	0.872	0.002	0.874	0.002	0.820	0.004	0.823	0.004

Supplementary Table 5. The significant differences that were observed for the ROC-AUC performance of the 20 ensemble BRT models trained on all incidence samples under different strategies.

Strategy	a	a+	b	b+
a	—	—	—	—
a+	9.804E-5 ***	—	—	—
b	1.451E-11***	1.451E-11***	—	—
b+	6.786E-8 ***	6.786E-8 ***	4.535E-6 ***	—

Note: * indicates $p<0.05$; ** indicates $p<0.01$; *** indicates $p<0.001$; NS indicates not significant; The p values were determined by two-tailed Mann–Whitney test, representing a comparison among strategies.

Supplementary Table 6. The significant differences that were observed for the ROC-AUC performance of the 20 ensemble BRT models trained on all onset samples under different strategies.

Strategy	a	a+	b	b+
a	—	—	—	—
a+	0.015 *	—	—	—
b	1.451E-11***	6.786E-8 ***	—	—
b+	1.451E-11***	6.786E-8 ***	2.898E-5 ***	—

Note: * indicates $p<0.05$; ** indicates $p<0.01$; *** indicates $p<0.001$; NS indicates not significant; The p values were determined by two-tailed Mann–Whitney test, representing a comparison among strategies.

Supplementary Table 7. The relative contribution of covariates in simulating the global risk of armed conflict incidence based on the 20 ensemble BRT models trained on all incidence samples from period 2000-2015 under strategies a and a+.

Variables	Relative contribution ± Standard Deviation, %	
	Strategy a	Strategy a+
Stable background covariates†	97.486	96.194
Mean temperature	46.493 ± 1.187	45.944 ± 1.171
Natural disaster hotspots	15.925 ± 0.725	15.706 ± 0.753
Mean precipitation	10.609 ± 0.885	10.545 ± 0.831
Urban accessibility	9.758 ± 0.667	9.684 ± 0.648
Elevation	5.900 ± 0.342	5.578 ± 0.293
Nighttime lights	3.150 ± 0.166	3.207 ± 0.197
Ethnic diversity	2.889 ± 0.191	2.835 ± 0.189
Normalized difference vegetation index	2.762 ± 0.197	2.695 ± 0.190
Climate deviations related covariates†	2.514	3.806
Standardized temperature index	1.720 ± 0.167	2.508 ± 0.231
Standardized precipitation index	0.794 ± 0.090	1.298 ± 0.133

Note: †Sum of relative contribution for both categories.

Supplementary Table 8. The relative contribution of covariates in simulating the global risk of armed conflict onset based on the 20 ensemble BRT models trained on all onset samples from period 2000-2015 under strategies a and a+.

Variables	Relative contribution ± Standard Deviation, %	
	Strategy a	Strategy a+
Stable background covariates†	96.894	96.067
Mean temperature	47.763 ± 1.667	47.158 ± 1.589
Natural disaster hotspots	14.267 ± 1.239	14.037 ± 1.206
Mean precipitation	10.896 ± 1.052	10.902 ± 1.057
Urban accessibility	9.901 ± 0.933	9.830 ± 0.928
Elevation	5.587 ± 0.370	5.293 ± 0.360
Ethnic diversity	3.093 ± 0.240	3.045 ± 0.214
Nighttime lights	2.815 ± 0.239	2.846 ± 0.244
Normalized difference vegetation index	2.572 ± 0.284	2.557 ± 0.272
Climate deviations related covariates†	3.106	4.331
Standardized temperature index	2.233 ± 0.314	2.955 ± 0.341
Standardized precipitation index	0.873 ± 0.092	1.376 ± 0.153

Note: †Sum of relative contribution for both categories.

Supplementary Notes

Dependent Variable

Data on armed conflict is taken from the openly available Uppsala Conflict Data Program (UCDP) georeferenced event dataset (GED). The UCDP GED is an armed conflict event dataset that includes state-based conflict, non-state conflict, and one-sided violence, and each armed conflict event is defined as: “an incident where armed force was used by an organised actor against another organized actor, or against civilians, resulting in at least 1 direct death at a specific location and a specific date” [1]. In order to alleviate the well-known media bias, UCDP GED does not rely solely on media reports, but also on NGO reports, case studies, databases and historical archives. In addition, triple-checked was employed to improve the quality of the final dataset. In contrast to most other event datasets, the quality of UCDP GED’s geocoding and precision information is much better [2], which is particularly important for us to analyze geographic dimensions of armed conflict. Therefore, UCDP GED was adopted in the present study. Based on UCDP GED, we aggregate armed conflict events to the grid-year level and code two binary dependent variables (armed conflict incidence and armed conflict onset) to represent the risk of armed conflict. The two indicators are coded using the following equation [1] and [2] [3]:

$$\text{Armed conflict incidence} = \begin{cases} 1 & \text{if armed conflict event in year } t \\ 0 & \text{if no armed conflict event in year } t \end{cases} \quad [1]$$

$$\text{Armed conflict onset} = \begin{cases} 1 & \text{if armed conflict event in year } t \text{ but not in year } t\text{-1} \\ 0 & \text{if no armed conflict event in year } t \text{ and year } t\text{-1} \end{cases} \quad [2]$$

Independent Variable

Previous studies have linked conflict risk to a series of covariates [4, 5]. For instance, politically relevant ethnic diversity might play a prominent role in conflict-prone regions, particularly in Africa and Asia, thus serving as a predetermined conflict line [6]. In addition, climate change could worsen instability in volatile regions, especially in Africa [7, 8, 9]. In the past decades, some interdisciplinary groups of scientists adopted various covariates to understand conflict risk and predict the risk of armed conflict; these primarily focused on a single country or region scale [10, 11, 12]. However, the grid-year level ($0.1° \times 0.1°$) prediction exercise at the global scale remains a huge challenge due to the complexity of the causal linkages and the availability of high-quality data. With an increasing number of various kinds of data and the further development of machine learning approaches, quantifying the causal effect of the climate-conflict link and making the grid-year level ($0.1° \times 0.1°$) prediction at the global scale have become possible. Considering the availability of data, several global fine-scale datasets described in Methods section were used to generate the candidate independent variables. The candidate independent variables adopted in the present study were divided into two categories: climate deviation related factors and stable background contexts. Climate deviation related factors included: (a) Standardized temperature index (One-year or Two-year); (b) Standardized precipitation index (One-year or Two-year). Stable background contexts included: (a) Mean temperature; (b) Mean precipitation; (c) Elevation; (d) Natural disaster hotspots; (e) Ethnic diversity; (f) Urban accessibility; (g) Nighttime lights; (h) Normalized difference vegetation index. The list of independent variables and statistical tests under different modelling strategies are detailed in chapters Modelling Strategy and Statistical Test respectively.

Supplementary Methods

Boosted Regression Trees

A comprehensive comparison of 16 modelling methods conducted by Elith et al. (2006) [13] revealed that boosted regression trees (BRT) and maximum entropy mode (Maxent) performed better than other modelling methods. Whilst broadly comparable, BRT tend to out-perform Maxent at capturing the complex relationships based on a large amount of data. Thus, BRT modelling framework was adopted in the present study.

The BRT model can be described using the following functional forms [3] and [4]:

$$f_t(X) = f_{t-1}(X) + \lambda \cdot \rho_t h(X; a_t) \qquad \lambda \in (0,1] \qquad [3]$$

$$L(y, f(X)) = \log(1 + \exp(-2yf(X))) \qquad [4]$$

where $X = \{x_1, x_2, \cdots, x_n\}$ represents stable background contexts and climate deviations related covariates, y is armed conflict incidence or armed conflict onset, $f_t(X)$ refers to the estimated mapping function from X to y during the t-th iteration process, λ is the learning-rate parameter, ρ_t is the weight parameter, $h(X; a_t)$ is the function of an individual tree, and a_t defines the split variables. During the modelling process, the parameters ρ_t and a_t were estimated by minimizing a binomial loss function (equation [4]).

In the present study, the R version 3.3.3 64-bit statistical computing platform was combined with the extension packages (i.e., dismo and gbm) to build BRT modelling framework and assess the performance. To enhance the robustness of simulation, we created an ensemble of 20 BRT models to generate the risk map of armed conflict using the mean method. Area under the receiver operator characteristic curve (ROC-AUC) [14, 15, 16], area under the precision recall curves (PR-AUC) and F_1-

score [11] were adopted as accuracy evaluation indexes.

BRT Parameter Selection

The following parameters are required to be determined during usage of BRT modelling framework: a) the complexity of individual trees (tree.complexity); b) the weight applied to individual trees (learning.rate); c) the proportion of observations used in selecting variables (bag.fraction); d) numbers of trees to add at each cycle (step.size); e) the number of folds cross-validation (cv.folds); f) max number of trees to fit before stopping (max.trees). For parameters a-f, we follow Bhatt et al. (2013) [17] in setting tree.complexity equal to 4, learning.rate equal to 0.01, bag.fraction equal to 0.75, step.size equal to 10, cv.folds equal to 10 and max.trees equal to 10000. In addition, it should be noted that specifying the optimal number of trees plays an important role during the BRT modelling process. In the present study, the methods of Elith et al. (2008) [18] was combined with 10-fold cross validation process to determine the optimal number of trees. Other parameters were held at their default values.

Modelling Strategy

In the present study, four modelling strategies (named strategies a, a+, b, b+, respectively) are designed to construct the dimensional information of samples. The detailed covariates adopted in the four modelling strategies are shown in Supplementary Table 9. The modelling process involves two stages, as shown in Supplementary Fig. 1. In the first stage, 20 ensemble BRT models trained on one-year incidence samples or one-year onset samples under four strategies are combined with time-cross validation method to prove the hypothesis that the patterns between conflict risk and high-dimensional covariates exist. In the second stage, the samples from 2000 to 2015 are merged

to form a larger sample, and the ensemble BRT models are trained on all incidence samples or all onset samples to avoid the models skew to the single-year sample. It should be noted that we also need to perform rigorous statistical tests on the data. The details of statistical tests are shown in the following section.

Supplementary Table 9. The stable background contexts and climate deviation related covariates adopted in the four modelling strategies.

Categories	Strategy a	Strategy a+	Strategy b	Strategy b+
Stable background contexts	Mean temperature<br>Natural disaster hotspots<br>Mean precipitation<br>Urban accessibility<br>Elevation<br>Nighttime lights<br>Ethnic diversity<br>Normalized difference vegetation index		Mean temperature<br>Mean precipitation	
Climate deviations related covariates	One-year standardized temperature index<br>One-year standardized precipitation index	Two-year standardized temperature index<br>Two-year standardized precipitation index	One-year standardized temperature index<br>One-year standardized precipitation index	Two-year standardized temperature index<br>Two-year standardized precipitation index

Statistical Test

If there is multicollinearity between covariate variables, unstable parameter estimates or other problems will decrease the credibility of the results revealed by the BRT model [19]. For the collinearity test, the correlation matrix and variance inflation factor (VIF) were calculated during two modelling processes. In the first stage, one-year incidence samples (2000) and one-year onset samples (2000) used in the first simulation process under four strategies are adopted to estimate correlation matrix and VIF, as shown in Supplementary Tables 10-17. In the second stage, all incidence samples and all onset samples under four strategies are adopted to estimate the correlation matrix and VIF, as shown in Supplementary Tables 18-25. Generally, these results illustrate that

multicollinearity is unlikely to affect our analysis.

Supplementary Table 10. Correlation matrix between covariate variables used in BRT ensembles trained on one-year (2000) incidence samples under strategy a.

	SPI	STI	E	NTL	UA	ED	NDH	MP	MT	NDVI
SPI	1	-0.304	-0.129	-0.053	0.087	-0.005	0.065	0.266	0.049	0.163
STI	-0.304	1	0.11	-0.059	-0.013	0.079	-0.053	-0.343	-0.031	-0.185
E	-0.129	0.11	1	-0.097	0.094	0.092	0.022	-0.135	-0.16	-0.169
NTL	-0.053	-0.059	-0.097	1	-0.171	0.107	0.008	0.007	0.105	0.046
UA	0.087	-0.013	0.094	-0.171	1	-0.295	-0.127	-0.184	-0.499	-0.36
ED	-0.005	0.079	0.092	0.107	-0.295	1	0.096	0.134	0.232	0.206
NDH	0.065	-0.053	0.022	0.008	-0.127	0.096	1	0.244	0.147	0.13
MP	0.266	-0.343	-0.135	0.007	-0.184	0.134	0.244	1	0.487	0.7
MT	0.049	-0.031	-0.16	0.105	-0.499	0.232	0.147	0.487	1	0.483
NDVI	0.163	-0.185	-0.169	0.046	-0.36	0.206	0.13	0.7	0.483	1

Note: SPI (Standardized precipitation index): VIF = 1.175; STI (Standardized temperature index): VIF = 1.260; E (Elevation): VIF = 1.091; NTL (Nighttime lights): VIF = 1.057; UA (Urban accessibility): VIF = 1.546; ED (Ethnic diversity): VIF = 1.161; NDH (Natural disaster hotspots) : VIF = 1.087; MP (Mean precipitation) : VIF = 2.607; MT (Mean temperature) : VIF = 1.768; NDVI (normalized difference vegetation index) : VIF = 2.273.

Supplementary Table 11. Correlation matrix between covariate variables used in BRT ensembles trained on one-year (2000) incidence samples under strategy a+.

	SPI	STI	E	NTL	UA	ED	NDH	MP	MT	NDVI
SPI	1	-0.384	-0.137	-0.059	0.043	0.006	0.084	0.363	0.16	0.223
STI	-0.384	1	0.149	0.002	-0.069	0.139	0.059	-0.333	-0.043	-0.232
E	-0.137	0.149	1	-0.097	0.094	0.092	0.022	-0.135	-0.16	-0.169
NTL	-0.059	0.002	-0.097	1	-0.171	0.107	0.008	0.007	0.105	0.046
UA	0.043	-0.069	0.094	-0.171	1	-0.295	-0.127	-0.184	-0.499	-0.36
ED	0.006	0.139	0.092	0.107	-0.295	1	0.096	0.134	0.232	0.206
NDH	0.084	0.059	0.022	0.008	-0.127	0.096	1	0.244	0.147	0.13
MP	0.363	-0.333	-0.135	0.007	-0.184	0.134	0.244	1	0.487	0.7
MT	0.16	-0.043	-0.16	0.105	-0.499	0.232	0.147	0.487	1	0.483
NDVI	0.223	-0.232	-0.169	0.046	-0.36	0.206	0.13	0.7	0.483	1

Note: SPI (Standardized precipitation index): VIF = 1.299; STI (Standardized temperature index): VIF = 1.342; E (Elevation): VIF = 1.092; NTL (Nighttime lights): VIF = 1.051; UA (Urban accessibility): VIF = 1.544; ED (Ethnic diversity): VIF = 1.176; NDH (Natural disaster hotspots): VIF = 1.107; MP (Mean precipitation): VIF = 2.567; MT (Mean temperature): VIF = 1.755; NDVI (normalized difference vegetation index): VIF = 2.275.

Supplementary Table 12. Correlation matrix between covariate variables used in BRT ensembles trained on one-year (2000) incidence samples under strategy b.

	SPI	STI	MP	MT
SPI	1	-0.304	0.266	0.049
STI	-0.304	1	-0.343	-0.031

MP	0.266	-0.343	1	0.487
MT	0.049	-0.031	0.487	1

Note: SPI (Standardized precipitation index): VIF = 1.143; STI (Standardized temperature index): VIF = 1.226; MP (Mean precipitation): VIF = 1.579; MT (Mean temperature): VIF = 1.352.

Supplementary Table 13. Correlation matrix between covariate variables used in BRT ensembles trained on one-year (2000) incidence samples under strategy b+.

	SPI	STI	MP	MT
SPI	1	-0.384	0.363	0.16
STI	-0.384	1	-0.333	-0.043
MP	0.363	-0.333	1	0.487
MT	0.16	-0.043	0.487	1

Note: SPI (Standardized precipitation index): VIF = 1.266; STI (Standardized temperature index): VIF = 1.262; MP (Mean precipitation): VIF = 1.581; MT (Mean temperature): VIF = 1.339.

Supplementary Table 14. Correlation matrix between covariate variables used in BRT ensembles trained on one-year (2000) onset samples under strategy a.

	SPI	STI	E	NTL	UA	ED	NDH	MP	MT	NDVI
SPI	1	-0.301	-0.148	-0.032	0.094	-0.012	0.095	0.24	0.016	0.146
STI	-0.301	1	0.058	-0.043	-0.017	0.064	-0.074	-0.371	-0.039	-0.213
E	-0.148	0.058	1	-0.114	0.029	0.112	0.006	-0.126	-0.156	-0.177
NTL	-0.032	-0.043	-0.114	1	-0.196	0.106	-0.008	-0.002	0.107	0.021
UA	0.094	-0.017	0.029	-0.196	1	-0.319	-0.14	-0.226	-0.539	-0.365
ED	-0.012	0.064	0.112	0.106	-0.319	1	0.137	0.129	0.212	0.193
NDH	0.095	-0.074	0.006	-0.008	-0.14	0.137	1	0.276	0.163	0.123
MP	0.24	-0.371	-0.126	-0.002	-0.226	0.129	0.276	1	0.495	0.703
MT	0.016	-0.039	-0.156	0.107	-0.539	0.212	0.163	0.495	1	0.485
NDVI	0.146	-0.213	-0.177	0.021	-0.365	0.193	0.123	0.703	0.485	1

Note: SPI (Standardized precipitation index): VIF = 1.170; STI (Standardized temperature index): VIF = 1.280; E (Elevation): VIF = 1.110; NTL (Nighttime lights): VIF = 1.074; UA (Urban accessibility): VIF = 1.648; ED (Ethnic diversity): VIF = 1.175; NDH (Natural disaster hotspots) : VIF = 1.125; MP (Mean precipitation) : VIF = 2.673; MT (Mean temperature) : VIF = 1.859; NDVI (normalized difference vegetation index) : VIF = 2.284.

Supplementary Table 15. Correlation matrix between covariate variables used in BRT ensembles trained on one-year (2000) onset samples under strategy a+.

	SPI	STI	E	NTL	UA	ED	NDH	MP	MT	NDVI
SPI	1	-0.396	-0.131	-0.071	0.054	-0.007	0.131	0.364	0.137	0.23
STI	-0.396	1	0.084	0.036	-0.094	0.113	0.047	-0.348	-0.057	-0.264
E	-0.131	0.084	1	-0.114	0.029	0.112	0.006	-0.126	-0.156	-0.177
NTL	-0.071	0.036	-0.114	1	-0.196	0.106	-0.008	-0.002	0.107	0.021
UA	0.054	-0.094	0.029	-0.196	1	-0.319	-0.14	-0.226	-0.539	-0.365
ED	-0.007	0.113	0.112	0.106	-0.319	1	0.137	0.129	0.212	0.193
NDH	0.131	0.047	0.006	-0.008	-0.14	0.137	1	0.276	0.163	0.123

MP	0.364	-0.348	-0.126	-0.002	-0.226	0.129	0.276	1	0.495	0.703
MT	0.137	-0.057	-0.156	0.107	-0.539	0.212	0.163	0.495	1	0.485
NDVI	0.23	-0.264	-0.177	0.021	-0.365	0.193	0.123	0.703	0.485	1

Note: SPI (Standardized precipitation index): VIF = 1.317; STI (Standardized temperature index): VIF = 1.356; E (Elevation): VIF = 1.101; NTL (Nighttime lights): VIF = 1.070; UA (Urban accessibility): VIF = 1.664; ED (Ethnic diversity): VIF = 1.180; NDH (Natural disaster hotspots): VIF = 1.149; MP (Mean precipitation): VIF = 2.603; MT (Mean temperature): VIF = 1.829; NDVI (normalized difference vegetation index): VIF = 2.301.

Supplementary Table 16. Correlation matrix between covariate variables used in BRT ensembles trained on one-year (2000) onset samples under strategy b.

	SPI	STI	MP	MT
SPI	1	-0.301	0.24	0.016
STI	-0.301	1	-0.371	-0.039
MP	0.24	-0.371	1	0.495
MT	0.016	-0.039	0.495	1

Note: SPI (Standardized precipitation index): VIF = 1.131; STI (Standardized temperature index): VIF = 1.258; MP (Mean precipitation): VIF = 1.630; MT (Mean temperature): VIF = 1.379.

Supplementary Table 17. Correlation matrix between covariate variables used in BRT ensembles trained on one-year (2000) onset samples under strategy b+.

	SPI	STI	MP	MT
SPI	1	-0.396	0.364	0.137
STI	-0.396	1	-0.348	-0.057
MP	0.364	-0.348	1	0.495
MT	0.137	-0.057	0.495	1

Note: SPI (Standardized precipitation index): VIF = 1.274; STI (Standardized temperature index): VIF = 1.279; MP (Mean precipitation): VIF = 1.621; MT (Mean temperature): VIF = 1.352.

Supplementary Table 18. Correlation matrix between covariate variables used in BRT ensembles trained on all incidence samples under strategy a.

	SPI	STI	E	NTL	UA	ED	NDH	MP	MT	NDVI
SPI	1	-0.106	0.022	-0.031	0.01	0.008	0.031	0.013	-0.03	0.026
STI	-0.106	1	0.01	-0.023	-0.037	-0.043	-0.005	-0.012	0.216	0.031
E	0.022	0.01	1	-0.108	0.06	0.111	0.002	-0.153	-0.155	-0.201
NTL	-0.031	-0.023	-0.108	1	-0.174	0.112	0.024	-0.009	0.11	0.027
UA	0.01	-0.037	0.06	-0.174	1	-0.285	-0.126	-0.138	-0.494	-0.261
ED	0.008	-0.043	0.111	0.112	-0.285	1	0.082	0.114	0.201	0.164
NDH	0.031	-0.005	0.002	0.024	-0.126	0.082	1	0.266	0.159	0.149
MP	0.013	-0.012	-0.153	-0.009	-0.138	0.114	0.266	1	0.419	0.736
MT	-0.03	0.216	-0.155	0.11	-0.494	0.201	0.159	0.419	1	0.372
NDVI	0.026	0.031	-0.201	0.027	-0.261	0.164	0.149	0.736	0.372	1

Note: SPI (Standardized precipitation index): VIF = 1.016; STI (Standardized temperature index): VIF = 1.098; E (Elevation): VIF = 1.099; NTL (Nighttime lights): VIF = 1.055; UA (Urban accessibility): VIF = 1.501; ED (Ethnic

diversity): VIF = 1.147; NDH (Natural disaster hotspots) : VIF = 1.100; MP (Mean precipitation) : VIF = 2.591; MT (Mean temperature) : VIF = 1.732; NDVI (normalized difference vegetation index) : VIF = 2.399.

Supplementary Table 19. Correlation matrix between covariate variables used in BRT ensembles trained on all incidence samples under strategy a+.

	SPI	STI	E	NTL	UA	ED	NDH	MP	MT	NDVI
SPI	1	-0.093	0.017	-0.046	0.02	0.004	0.044	0.043	-0.022	0.051
STI	-0.093	1	0.022	-0.019	-0.038	-0.043	-0.007	-0.028	0.239	0.02
E	0.017	0.022	1	-0.108	0.06	0.111	0.002	-0.153	-0.155	-0.201
NTL	-0.046	-0.019	-0.108	1	-0.174	0.112	0.024	-0.009	0.11	0.027
UA	0.02	-0.038	0.06	-0.174	1	-0.285	-0.126	-0.138	-0.494	-0.261
ED	0.004	-0.043	0.111	0.112	-0.285	1	0.082	0.114	0.201	0.164
NDH	0.044	-0.007	0.002	0.024	-0.126	0.082	1	0.266	0.159	0.149
MP	0.043	-0.028	-0.153	-0.009	-0.138	0.114	0.266	1	0.419	0.736
MT	-0.022	0.239	-0.155	0.11	-0.494	0.201	0.159	0.419	1	0.372
NDVI	0.051	0.02	-0.201	0.027	-0.261	0.164	0.149	0.736	0.372	1

Note: SPI (Standardized precipitation index): VIF = 1.017; STI (Standardized temperature index): VIF = 1.122; E (Elevation): VIF = 1.101; NTL (Nighttime lights): VIF = 1.056; UA (Urban accessibility): VIF = 1.506; ED (Ethnic diversity): VIF = 1.147; NDH (Natural disaster hotspots): VIF = 1.100; MP (Mean precipitation): VIF = 2.607; MT (Mean temperature): VIF = 1.772; NDVI (normalized difference vegetation index): VIF = 2.402.

Supplementary Table 20. Correlation matrix between covariate variables used in BRT ensembles trained on all incidence samples under strategy b.

	SPI	STI	MP	MT
SPI	1	-0.106	0.013	-0.03
STI	-0.106	1	-0.012	0.216
MP	0.013	-0.012	1	0.419
MT	-0.03	0.216	0.419	1

Note: SPI (Standardized precipitation index): VIF = 1.012; STI (Standardized temperature index): VIF = 1.074; MP (Mean precipitation): VIF = 1.229; MT (Mean temperature): VIF = 1.289.

Supplementary Table 21. Correlation matrix between covariate variables used in BRT ensembles trained on all incidence samples under strategy b+.

	SPI	STI	MP	MT
SPI	1	-0.093	0.043	-0.022
STI	-0.093	1	-0.028	0.239
MP	0.043	-0.028	1	0.419
MT	-0.022	0.239	0.419	1

Note: SPI (Standardized precipitation index): VIF = 1.011; STI (Standardized temperature index): VIF = 1.091; MP (Mean precipitation): VIF = 1.241; MT (Mean temperature): VIF = 1.313.

Supplementary Table 22. Correlation matrix between covariate variables used in BRT

ensembles trained on all onset samples under strategy a.

	SPI	STI	E	NTL	UA	ED	NDH	MP	MT	NDVI
SPI	1	-0.092	0.005	-0.027	0.013	0.012	0.03	0.006	-0.025	0.035
STI	-0.092	1	0.021	-0.027	-0.051	-0.03	-0.021	-0.025	0.219	0.03
E	0.005	0.021	1	-0.098	0.044	0.111	-0.017	-0.146	-0.152	-0.187
NTL	-0.027	-0.027	-0.098	1	-0.178	0.096	0.022	-0.003	0.099	0.029
UA	0.013	-0.051	0.044	-0.178	1	-0.279	-0.131	-0.139	-0.504	-0.262
ED	0.012	-0.03	0.111	0.096	-0.279	1	0.11	0.116	0.18	0.159
NDH	0.03	-0.021	-0.017	0.022	-0.131	0.11	1	0.304	0.172	0.171
MP	0.006	-0.025	-0.146	-0.003	-0.139	0.116	0.304	1	0.425	0.727
MT	-0.025	0.219	-0.152	0.099	-0.504	0.18	0.172	0.425	1	0.376
NDVI	0.035	0.03	-0.187	0.029	-0.262	0.159	0.171	0.727	0.376	1

Note: SPI (Standardized precipitation index): VIF = 1.014; STI (Standardized temperature index): VIF = 1.099; E (Elevation): VIF = 1.089; NTL (Nighttime lights): VIF = 1.050; UA (Urban accessibility): VIF = 1.530; ED (Ethnic diversity): VIF = 1.133; NDH (Natural disaster hotspots) : VIF = 1.129; MP (Mean precipitation) : VIF = 2.590; MT (Mean temperature) : VIF = 1.765; NDVI (normalized difference vegetation index) : VIF = 2.339.

Supplementary Table 23. Correlation matrix between covariate variables used in BRT ensembles trained on all onset samples under strategy a+.

	SPI	STI	E	NTL	UA	ED	NDH	MP	MT	NDVI
SPI	1	-0.073	-0.005	-0.042	0.02	0.007	0.05	0.045	-0.01	0.069
STI	-0.073	1	0.028	-0.017	-0.056	-0.031	-0.025	-0.042	0.237	0.02
E	-0.005	0.028	1	-0.098	0.044	0.111	-0.017	-0.146	-0.152	-0.187
NTL	-0.042	-0.017	-0.098	1	-0.178	0.096	0.022	-0.003	0.099	0.029
UA	0.02	-0.056	0.044	-0.178	1	-0.279	-0.131	-0.139	-0.504	-0.262
ED	0.007	-0.031	0.111	0.096	-0.279	1	0.11	0.116	0.18	0.159
NDH	0.05	-0.025	-0.017	0.022	-0.131	0.11	1	0.304	0.172	0.171
MP	0.045	-0.042	-0.146	-0.003	-0.139	0.116	0.304	1	0.425	0.727
MT	-0.01	0.237	-0.152	0.099	-0.504	0.18	0.172	0.425	1	0.376
NDVI	0.069	0.02	-0.187	0.029	-0.262	0.159	0.171	0.727	0.376	1

Note: SPI (Standardized precipitation index): VIF = 1.016; STI (Standardized temperature index): VIF = 1.119; E (Elevation): VIF = 1.090; NTL (Nighttime lights): VIF = 1.051; UA (Urban accessibility): VIF = 1.533; ED (Ethnic diversity): VIF = 1.133; NDH (Natural disaster hotspots): VIF = 1.130; MP (Mean precipitation): VIF = 2.606; MT (Mean temperature): VIF = 1.799; NDVI (normalized difference vegetation index): VIF = 2.344.

Supplementary Table 24. Correlation matrix between covariate variables used in BRT ensembles trained on all onset samples under strategy b.

	SPI	STI	MP	MT
SPI	1	-0.092	0.006	-0.025
STI	-0.092	1	-0.025	0.219
MP	0.006	-0.025	1	0.425
MT	-0.025	0.219	0.425	1

Note: SPI (Standardized precipitation index): VIF = 1.009; STI (Standardized temperature index): VIF = 1.077; MP (Mean precipitation): VIF = 1.242; MT (Mean temperature): VIF = 1.304.

Supplementary Table 25. Correlation matrix between covariate variables used in BRT ensembles trained on all onset samples under strategy b+.

	SPI	STI	MP	MT
SPI	1	-0.073	0.045	-0.01
STI	-0.073	1	-0.042	0.237
MP	0.045	-0.042	1	0.425
MT	-0.01	0.237	0.425	1

Note: SPI (Standardized precipitation index): VIF = 1.007; STI (Standardized temperature index): VIF = 1.093; MP (Mean precipitation): VIF = 1.256; MT (Mean temperature): VIF = 1.326.

Supplementary References

1. Sundberg R, Melander E. Introducing the UCDP georeferenced event dataset. *Journal of Peace Research* **50**, 523-532 (2013).
2. Eck, K. In data we trust? A comparison of UCDP GED and ACLED conflict events datasets. *Cooperation & Conflict* **47**, 124-141 (2012).
3. Van Weezel S. Economic shocks & civil conflict onset in Sub-Saharan Africa, 1981–2010. *Defence and Peace Economics* **26**, 153-177 (2015).
4. Uexkull VN, Croicu M, Fjelde H, Buhaug H. Civil conflict sensitivity to growing-season drought. *Proceedings of the National Academy of Sciences* **113**, 12391-12396 (2016).
5. Hsiang SM, Burke M, Miguel E. Quantifying the Influence of Climate on Human Conflict. *Science* **341**, 1235367 (2013).
6. Schleussner CF, Donges JF, Donner RV, Schellnhuber HJ. Armed-conflict risks enhanced by climate-related disasters in ethnically fractionalized countries. *Proceedings of the National Academy of Sciences* **113**, 9216-9221 (2016).
7. O'Loughlin J, Witmer FD, Linke AM, Laing A, Gettelman A, Dudhia J. Climate variability and conflict risk in East Africa, 1990–2009. *Proceedings of the National Academy of Sciences* **109**, 18344-18349 (2012).
8. Burke MB, Miguel E, Satyanath S, Dykema JA, Lobell DB. Warming increases the risk of civil war in Africa. *Proceedings of the National Academy of Sciences of the United States of America* **106**, 20670-20674 (2009).
9. Barnaby W. Do nations go to war over water? *Nature* **458**, 282-283 (2009).
10. O'Loughlin J, Linke AM, Witmer FD. Effects of temperature and precipitation variability on the risk of violence in sub-Saharan Africa, 1980–2012. *Proceedings of the National Academy of Sciences* **111**, 16712-16717 (2014).
11. Hegre H*, et al.* ViEWS: A political violence early-warning system. *Journal of Peace Research* **56**, 155-174 (2019).
12. Bazzi S, Blair R, Blattman C, Dube O, Gudgeon M, Peck R. The Promise and Pitfalls of Conflict Prediction: Evidence from Colombia and Indonesia. *The Review of Economics and*

Statistics, 1-45 (2021).

13. Elith J*, et al.* Novel methods improve prediction of species' distributions from occurrence data. *Ecography* **29**, 129-151 (2006).

14. Ding F, Ge Q, Jiang D, Fu J, Hao M. Understanding the dynamics of terrorism events with multiple-discipline datasets and machine learning approach. *PloS one* **12**, e0179057 (2017).

15. Hao M, Jiang D, Ding F, Fu J, Chen S. Simulating spatio-temporal patterns of terrorism incidents on the indochina peninsula with GIS and the random forest method. *ISPRS International Journal of Geo-Information* **8**, 133 (2019).

16. Messina JP*, et al.* The current and future global distribution and population at risk of dengue. *Nature microbiology* **4**, 1508-1515 (2019).

17. Bhatt S*, et al.* The global distribution and burden of dengue. *Nature* **496**, 504-507 (2013).

18. Elith J, Leathwick JR, Hastie T. A working guide to boosted regression trees. *Journal of Animal Ecology* **77**, 802-813 (2008).

19. Dormann CF*, et al.* Collinearity: a review of methods to deal with it and a simulation study evaluating their performance. *Ecography* **36**, 27-46 (2013).
