# Supplementary material for: Modelling armed conflict risk under climate change with machine learning and time-series data
Source: Nat Commun. 2022 May 20;13:2839. doi: 10.1038/s41467-022-30356-x (PMC9123163; doi:10.1038/s41467-022-30356-x)
Supplement: Supplementary file 3 — Reporting Summary [file 41467_2022_30356_MOESM3_ESM.pdf]

nature research

Corresponding author(s): Fangyu Ding, Dong Jiang

Last updated by author(s): Mar 15, 2022

Reporting Summary

Nature Research wishes to improve the reproducibility of the work that we publish. This form provides structure for consistency and transparency in reporting. For further information on Nature Research policies, see our Editorial Policies and the Editorial Policy Checklist.

Statistics

For all statistical analyses, confirm that the following items are present in the figure legend, table legend, main text, or Methods section.

n/a	Confirmed	
☐	☒	The exact sample size (n) for each experimental group/condition, given as a discrete number and unit of measurement
☒	☐	A statement on whether measurements were taken from distinct samples or whether the same sample was measured repeatedly
☐	☒	The statistical test(s) used AND whether they are one- or two-sided<br>*Only common tests should be described solely by name; describe more complex techniques in the Methods section.*
☐	☒	A description of all covariates tested
☐	☒	A description of any assumptions or corrections, such as tests of normality and adjustment for multiple comparisons
☐	☒	A full description of the statistical parameters including central tendency (e.g. means) or other basic estimates (e.g. regression coefficient) AND variation (e.g. standard deviation) or associated estimates of uncertainty (e.g. confidence intervals)
☐	☒	For null hypothesis testing, the test statistic (e.g. F, t, r) with confidence intervals, effect sizes, degrees of freedom and P value noted<br>*Give P values as exact values whenever suitable.*
☒	☐	For Bayesian analysis, information on the choice of priors and Markov chain Monte Carlo settings
☒	☐	For hierarchical and complex designs, identification of the appropriate level for tests and full reporting of outcomes
☒	☐	Estimates of effect sizes (e.g. Cohen's d, Pearson's r), indicating how they were calculated

Our web collection on statistics for biologists contains articles on many of the points above.

Software and code

Policy information about availability of computer code

Data collection	No software was used.
Data analysis	R software (Version X64 3.3.3). The codes used in the present study are freely available online at https://github.com/Celyon/ConflictRisk. The extension packages (i.e., dismo and gbm) were used to build the modelling framework and assess the performance.

For manuscripts utilizing custom algorithms or software that are central to the research but not yet described in published literature, software must be made available to editors and reviewers. We strongly encourage code deposition in a community repository (e.g. GitHub). See the Nature Research guidelines for submitting code & software for further information.

Data

Policy information about availability of data

All manuscripts must include a data availability statement. This statement should provide the following information, where applicable:

- Accession codes, unique identifiers, or web links for publicly available datasets
- A list of figures that have associated raw data
- A description of any restrictions on data availability

All data used in this study is publicly available. UCDP GED 17.1 version can be accessed from https://pcr.uu.se/research/ucdp/. The Climate Research Unit TS4.0 global dataset can be accessed from https://www.uea.ac.uk/web/groups-and-centres/climatic-research-unit/. The AVHRR normalized difference vegetation index dataset can be accessed from https://ecocast.arc.nasa.gov/data/pub/gimms/. Natural Disaster Hotspots dataset can be accessed from https://sedac.ciesin.columbia.edu/. Elevation dataset can be accessed from https://eospso.gsfc.nasa.gov/missions/shuttle-radar-topography-mission. The GeoEPR 2014 dataset can be accessed from https://icr.ethz.ch/. The urban accessibility dataset is available on https://forobs.jrc.ec.europa.eu/products/gam/. Nighttime lights dataset was downloaded from https://ngdc.noaa.gov/. The source data that support the findings of this study are available at Figshare (DOI: https://doi.org/10.6084/m9.figshare.15147333.v1).

Field-specific reporting

Please select the one below that is the best fit for your research. If you are not sure, read the appropriate sections before making your selection.

☐ Life sciences ☐ Behavioural & social sciences ☒ Ecological, evolutionary & environmental sciences

For a reference copy of the document with all sections, see nature.com/documents/nr-reporting-summary-flat.pdf

Ecological, evolutionary & environmental sciences study design

All studies must disclose on these points even when the disclosure is negative.

Study description	In this study, we adopted a formal machine learning-based modelling framework to infer potential causal linkages from high-frequency time-series data and simulate the risk of armed conflict worldwide from 2000 to 2015.
Research sample	This study is based on the most comprehensive armed conflict georeferenced event dataset (GED), which is taken from the openly available Uppsala Conflict Data Program (UCDP). In contrast to most other armed conflict event datasets, the quality of UCDP GED's geocoding and information precision is much better, which is particularly important for us to measure the frequency of the onset and incidence of armed conflict events in spatial and time units. However, UCDP was unable to resolve the bias in the GED completely and to include all armed conflict events in its dataset, which may add uncertainty to our results to some extent.
Sampling strategy	Based on UCDP GED, we aggregate armed conflict events to the grid-year level and code two binary dependent variables (armed conflict incidence and armed conflict onset) to represent the risk of armed conflict. If there are one or more instances of armed conflict event in one grid in a single year, the armed conflict incidence indicator is coded as one (high-risk) for the grid. In addition, if a new armed conflict event outbreak occurs after one calendar year of inactivity in one grid, armed conflict onset is assigned the value of one for this grid. Both binary dependent variables are otherwise assigned the value of zero (low-risk). For each year, an equivalent amount of low-risk samples and high-risk samples are randomly selected to construct the one-year samples and to train the boosted regression tree (BRT) model under the R language programming platform. Once the amount of data is insufficient to build an appropriate model, the programming platform will terminate the process of building the model and prompt that the amount of data is insufficient. In this study, we did not encounter this situation in the process of building the model, which suggests that these sample sizes are sufficient.
Data collection	Various data collection methods were used in this study and are detailed in the Methods.
Timing and spatial scale	Time: From 2000-2015.<br>Space: Global
Data exclusions	No data was excluded.
Reproducibility	These experiments run independently on two servers and were replicated successfully a minimum of 2 times.
Randomization	For each year, an equivalent amount of low-risk samples and high-risk samples are randomly selected to construct the one-year samples. The process of randomly selecting low-risk samples is realized through set.seed() and sample() functions under the R language programming platform.
Blinding	Blinding was not relevant to this study.

Did the study involve field work? ☐ Yes ☒ No

Reporting for specific materials, systems and methods

We require information from authors about some types of materials, experimental systems and methods used in many studies. Here, indicate whether each material, system or method listed is relevant to your study. If you are not sure if a list item applies to your research, read the appropriate section before selecting a response.

Materials & experimental systems

n/a	Involved in the study
☒	☐ Antibodies
☒	☐ Eukaryotic cell lines
☒	☐ Palaeontology and archaeology
☒	☐ Animals and other organisms
☒	☐ Human research participants
☒	☐ Clinical data
☒	☐ Dual use research of concern

Methods

n/a	Involved in the study
☒	☐ ChIP-seq
☒	☐ Flow cytometry
☒	☐ MRI-based neuroimaging
